# Amniotic Membrane Modifies the Genetic Program Induced by TGFß, Stimulating Keratinocyte Proliferation and Migration in Chronic Wounds

**DOI:** 10.1371/journal.pone.0135324

**Published:** 2015-08-18

**Authors:** Antonia Alcaraz, Anna Mrowiec, Carmen Luisa Insausti, Ángel Bernabé-García, Eva María García-Vizcaíno, María Concepción López-Martínez, Asunción Monfort, Ander Izeta, José María Moraleda, Gregorio Castellanos, Francisco José Nicolás

**Affiliations:** 1 Oncología Molecular y TGFß, Unidad de Investigación, Hospital Universitario Virgen de la Arrixaca, El Palmar, Murcia, Spain; 2 Unidad de Terapia Celular, Hospital Clínico Universitario Virgen de la Arrixaca, El Palmar, Murcia, Spain; 3 Instituto Biodonostia, Hospital Universitario Donostia, San Sebastian, Spain; 4 Servicio de Cirugía, Hospital Universitario Virgen de la Arrixaca, El Palmar, Murcia, Spain; National Centre for Scientific Research, 'Demokritos', GREECE

## Abstract

**Background:**

Post-traumatic large-surface or deep wounds often cannot progress to reepithelialisation because they become irresponsive in the inflammatory stage, so intervention is necessary to provide the final sealing epidermis. Previously we have shown that Amniotic Membrane (AM) induced a robust epithelialisation in deep traumatic wounds.

**Methods and Findings:**

To better understand this phenomenon, we used keratinocytes to investigate the effect of AM on chronic wounds. Using keratinocytes, we saw that AM treatment is able to exert an attenuating effect upon Smad2 and Smad3 TGFß-induced phosphorylation while triggering the activation of several MAPK signalling pathways, including ERK and JNK1, 2. This also has a consequence for TGFß-induced regulation on cell cycle control key players CDK1A (p21) and CDK2B (p15). The study of a wider set of TGFß regulated genes showed that the effect of AM was not wide but very concrete for some genes. TGFß exerted a powerful cell cycle arrest; the presence of AM however prevented TGFß-induced cell cycle arrest. Moreover, AM induced a powerful cell migration response that correlates well with the expression of c-Jun protein at the border of the healing assay. Consistently, the treatment with AM of human chronic wounds induced a robust expression of c-Jun at the wound border.

**Conclusions:**

The effect of AM on the modulation of TGFß responses in keratinocytes that favours proliferation together with AM-induced keratinocyte migration is the perfect match that allows chronic wounds to move on from their non-healing state and progress into epithelialization. Our results may explain why the application of AM on chronic wounds is able to promote epithelialisation.

## Introduction

Wound healing is the body’s natural biological process for regenerating dermal and epidermal tissue, which involves a delicate balanced activity of inflammatory, vascular, connective tissue and epithelial cells [1].

Acute wounds heal rapidly and proceed through the inflammatory, proliferation and remodelling phases of healing. Re-epithelialisation is the final and very important phase that occurs through the migration of keratinocytes from the edge toward the wound centre. Large-surface or deep wounds, with an important loss of soft tissues, often become senescent in the inflammatory or proliferation stages and cannot progress to re-epithelialisation [1, 2]. This failure in the re-epithelialisation process requires the need for intervention in order to provide the epithelial layer for the final sealing of the skin [1, 2]. Recently, the usage of Amniotic Membrane (AM) has proven a very effective way of overcoming the lack of epithelial proliferation due to the senescence and inflammation observed in chronic large wounds [3].

The AM is a tissue of particular interest due to its biological properties and immunologic characteristics. AM, the most internal layer of foetal membranes, consists of a thin epithelium, a basal membrane and a stroma of avascular connective tissue. Both epithelial and mesenchymal amniotic cells possess characteristics of stem cells with, at least, multi-potent differentiation ability, which makes AM a good candidate for use in cellular therapy and regenerative medicine [4, 5]. AM has low immunogenicity, and well-documented re-epithelialisation effects, as well as anti-inflammatory, anti-fibrotic, anti-microbial, and non-tumorigenic properties. These pleiotropic functions are related in part to its capacity to synthesize and release biological active substances including cytokines and signalling molecules such as the Tumour Necrosis Factor (TNFα), Interferon, Transforming Growth Factor α (TGFα), Transforming Growth Factor β (TGFβ), Epidermal Growth Factor (EGF), Keratinocyte Growth Factor (KGF), several interleukins, and Prostaglandins, among others [5–7].

In recent years there has been a resurgence of interest in AM and amnion transplantation because of its ability to enhance wound healing by promoting re-epithelialisation and reducing scarring and inflammation [8]. Also, AM has been used in ophthalmology [8–11] and in the treatment of non-healing ulcers of different aetiologies [3, 12] with satisfactory results. Our hospital has pioneered the application of AM in large deep extensive wounds obtaining promising results in the treatment and clinical management of these kinds of wounds. AM application was capable of restoring skin integrity avoiding the need for skin graft reconstruction [3]. Despite all these applications, uncertainty remains regarding the molecular effectors responsible for AM effects.

The mechanisms involved in AM induced skin re-epithelialisation are largely unknown. In our lab we have been using HaCaT cells, a spontaneously immortalized human keratinocyte cell line, as a model to understand the molecular consequences of the application of AM to human wounds. We have shown that HaCaT cells exhibited different molecular responses upon stimulation with AM that were attributed to the effects of soluble AM-released factors on HaCaT cells [3]. Additionally, HaCaT cells stimulated with AM showed an increased expression of *c-JUN*. Members of the AP1 family had been involved in the keratinocyte migration and wound healing process [13–16]. AM induced the phosphorylation of JNK1 and 2 kinases in HaCaT cells; JNK1 is a positive regulator of c-Jun, it contributes to its phosphorylation and stabilization [17, 18].

TGFß plays a critical role in regulating multiple cellular responses that occur in all three phases of wound healing [19]. Of the many cytokines shown to influence the wound healing process, TGFß has the broadest spectrum of action [20]. TGFß affects the behaviour of a wide variety of cell types and mediates a diverse range of cellular functions [20]. The TGFß signalling pathway is considered a promising target for the treatment of many pathological skin conditions including chronic non-healing wounds [19]. Platelets are thought to be the primary source of TGFß at the wound site; also, activation of latent TGFß occurs immediately after wounding [19].

TGFß1 is found at very high levels in the wound microenvironment and promotes myofibroblast differentiation, the production of extracellular matrix (ECM) components, and fibroblast chemotaxis [21]. Keratinocytes, fibroblasts and monocytes are among the targeted cells at the TGFß management of the wound [20]. Monocytes/macrophages and fibroblasts then act autocrinally to perpetuate high concentrations of TGFß at the wound [20].

TGFβ exerts its cellular effects by binding to heterotetrameric complexes of type I and type II serine/threonine kinase receptors. Upon formation of these complexes, the constitutively active type II receptor phosphorylates and activates the type I receptor [22]. Important substrates for the type I TGFβ receptor are members of the receptor activated (R-) Smad family (Smad2 and 3), although non-Smad pathways are also activated, including the extracellular-signal-regulated kinase (ERK), c-Jun N-terminal kinase (JNK), p38 mitogen-activated protein (MAP) kinase pathways, the tyrosine kinase Src and phosphatidylinositol 3'-kinase (PI3K) [23, 24]. After receptor-induced phosphorylation, R-Smads form complexes with the common-mediator (Co-) Smad4, which regulate the transcription of many genes [25].

TGFß causes the growth arrest of epithelial cells. The mechanisms involve the inhibition of the expression of some transcription factors such as Id family members and Myc, and the induction of the cell cycle inhibitors CDKN2B (p15) and CDKN1A (p21) [26]. The downregulation of Smad3 has been suggested as a possible way of improving wound healing [20]. Notably, the ability of keratinocytes to sense TGFß through Smad3 prevents the cell proliferation of keratinocytes and consequently prevents wound healing resolution when TGFß levels are high [27].

The effects of TGFß on full-thickness wound reepithelialisation have been studied. The usage of transgenic mice with overexpressed TGFß at the epidermis level shows a decrease in reepithelialisation [28, 29]. The study in the ear mouse model suggests that TGFß has an inhibitory effect on epithelialisation when the wound involves all the layers of the skin [30]. Abolishing part of the TGFß signalling pathway has been suggested as a solution for improving wound healing, so abolishing part of the TGFß stimulated Smad pathways may improve wound healing and attenuate the effect of TGFß signalling over fibroblast matrix synthesis, for instance [20]. In addition, the use of Smad3 antisense oligonucleotides accelerated wound healing and reduced scarring in a mouse excisional wound model [31].

Here, we show that AM may improve wound healing in massive wounds by antagonising some of the TGFß signalling effects. We also show that AM modifies the genetic program of keratinocytes affecting TGFß-regulated cell cycle arrest and improving cell migration.

## Materials and Methods

This study was approved by the local ethics committee (Virgen de la Arrixaca University Clinical Hospital, Murcia, Spain), the Spanish Agency for Drugs (AEMPS), and was conducted after appropriate written informed consent was obtained from the AM donors and patients treated with AM.

### HaCaT and primary keratinocyte cell-culture

Human spontaneously immortalized keratinocytes (HaCaT) [32] were grown in Dulbecco’s Modified Eagle Medium (DMEM) (Biowest, Nuaillé, France). Mink Lung Epithelial cells (Mv1Lu) [33–35] were grown in Eagles’s Minimum Essential Medium (EMEM) (Biowest, Nuaillé, France). Both media were supplemented with 50 U/ml penicillin, 50 μg/ml streptomycin (P/S) (Sigma-Aldrich, St Louis, MO, USA) and 10% Foetal Bovine Serum (FBS) (Thermo Fisher Scientific, Waltham, MA USA). Inhibitors and cytokines were used at the following concentrations: 10 ng/ml hEGF, 15 μM SP600125, 1 μM SB203580, 50 μM PD98059, 10 μM U0126 (all Sigma-Aldrich, St Louis, MO, USA), 2 ng/ml TGFβ (PeproTech, Rocky Hill, NJ, USA).

Human primary keratinocytes were obtained from healthy subjects undergoing plastic or circumcision surgery in the Policlínica Gipuzkoa or University Hospital, Donostia. Skin biopsies (surgical remnants) were obtained from donors who gave informed consent, after protocol approval by the relevant Clinical Research & Ethical Committees. Informed consent was obtained from parents when donors were under the age of 12. The isolation and culture of primary human keratinocytes was as follows: primary human skin cells were obtained from healthy subjects undergoing plastic or circumcision surgery. All biopsies were stored in RPMI-1640 medium (Sigma-Aldrich, St Louis, MO, USA) supplemented with 2% P/S (Sigma-Aldrich, St Louis, MO, USA). They were processed at a maximum 24h post-surgery. Keratinocytes were obtained and expanded from whole-thickness skin samples. Briefly, skin was chopped and disaggregated in 0.25% trypsin-EDTA (Sigma-Aldrich, St Louis, MO, USA) solution in three digestions of 45 min, 30 min and 30 min, at 37°C, under agitation. Cells were filtered through a 40 μm cell strainer (Beckton Dickinson, Franklin Lakes, NJ, USA) and centrifuged for 10 min at 1,500 rpm at RT. Keratinocytes were seeded at a density of 25,000–30,000 cells/cm^2^ on a lethally irradiated (50 Gy) 3T3 feeder layer seeded at a density of 75,000–80,000 cells/cm^2^. Keratinocyte culture medium was a 3:1 mixture of DMEM (with Glutamax, Gibco, Thermo Fisher Scientific, Waltham, MA USA) and Ham’s F12 (Sigma-Aldrich, St Louis, MO, USA) media, supplemented with 10% FBS, 100 μg/ml P/S, 0.18 mM Adenine (Sigma-Aldrich, St Louis, MO, USA), 2 nM 3,3´,5-Triyodo-L-tironina (Fluka, Sigma-Aldrich, St Louis, MO, USA), 0.4 μg/ml hydrocortisone (Sigma-Aldrich, St Louis, MO, USA), 0.1 nM Cholera toxin (Gentaur, Kampenhout, Belgium), 5 μg/ml bovine insulin (Sigma-Aldrich, St Louis, MO, USA) and 10 ng/ml epidermal growth factor (EGF) (Austral Biologicals, San Ramon, CA, USA). Media were changed every other day. Before assaying, 0.02% EDTA (Sigma-Aldrich, St Louis, MO, USA) was added to remove 3T3 feeder layer cells. Then the keratinocytes that were remaining were washed twice with PBS and the keratinocyte media was added again and cells were incubated at 37°C for 1 h before initiating the experiment.

### AM assay on cells

To perform the AM assay, 5 to 6 pieces of 4 cm^2^ of AM were placed in 6 cm diameter dishes containing 60% to 70% confluent HaCaT or primary keratinocyte cells. Cells stimulated for several periods of time with AM and/or TGFβ (PeproTech, Rocky Hill, NJ, USA) (2ng/ml for HaCaT cells or 30 ng/ml for human primary keratinocytes), were lysed using the appropriate buffers, then, either protein or RNA was extracted. In the case of protein studies, cells were lysed with 20 mM HCl-Tris pH 7.5, 150 mM NaCl, 5 mM EDTA, 1.2 mM MgCl_2_, 10% Glycerol, 0.5% NP 40 (Igepal), 1 mM DTT, 25 mM NaF and 25 mM β-glycerophosphate (all from Sigma-Aldrich, St Louis, MO, USA) supplemented with 10 mM Sodium Butyrate, phosphatase inhibitors (I and II) and protease inhibitors (all from Sigma-Aldrich, St Louis, MO, USA). Protein extracts were analysed by SDS-PAGE followed by western blot using the appropriate antibodies. As loading control we used ZO-1, Grb2 or ß-actin, alternately after checking that all of them did not experience any changes in expression upon TGFß, AM or both treatments together.

**Table 1:** Primers used for quantitative PCR.

Sequence 5’ to 3’	Name of the primer
GGAAACGACCTTCTATGACGATGCCC	c-JUN-F
GGCGCGCACGAAGCCCTCGGCGAACC	c-JUN-R
CATGGGGCCATGGAACAAGG	PAI1-F
CTTCCTGAGGTCGACTTCAG	PAI1-R
GATGCCGCGCTCCTTCCTGGTC	SNAI2-F
GCTGCTTATGTTTGGCCAGCC	SNAI2-R
ATGTCAGAACCGGCTGGGGATG	CDKN1A-F
GGGCTTCCTCTTGGAGAAGATC	CDKN1A-R
GCTGGCGACCACAAGGACCC	AKAP12-F
CTTGCTCCTCTGAGGGCAGC	AKAP12-R
GAATGCGCGAGGAGAACAAGGG	CDKN2B-F
CGTCAGTCCCCCGTGGCTGTG	CDKN2B-R
CTCCGCCTCCATGGATGACG	ITGB6-F
CCAAGACAGTTGACATGGAG	ITGB6-R
CCTTAGCCGACTCTGCGAACTA	MADH7-F
TGCATAAACTCGTGGTCATTGG	MADH7-R
ATGATGGTGATGGTGGTGGTG	TMEPAI-F
CTATCCATCAGGTCACTGTCG	TMEPAI-R
ACCACAGTCCATGCCATCAC	GAPDH-F
TCCACCACCCTGTTGCTGTA	GAPDH-R

For gene expression estimation, RNA was extracted using RNeasy-mini (Qiagen, Venlo, The Nederlands). Typically, 1 μg of RNA was retro-transcribed by GeneAmp (AppliedBiosystems, Thermo Fisher Scientific, Waltham, MA USA) or iScript (Bio-Rad, Hercules, CA, USA), and resulting cDNA was used for qPCR using the SYBR premix ex Taq kit (Takara Bio Europe/Clontech, Saint-Germain-en-Laye, France) according to the manufacturer’s instructions. Two samples of three independent experiments were quantified by qPCR. Gene expression levels were normalized to Glyceraldehyde 3-phosphate dehydrogenase (*GAPDH*) mRNA levels and data represent mean ± SEM. Gene expression values were represented as a fold increase in comparison to the control sample. Data were analyzed by unpaired two-tailed Student’s *t*-test to determine differences between the samples using Graph Pad application software. At the figure legends, the asterisks denote statistically significant differences between the treatments according to Student’s *t*-test. *p<0.05, **p<0.005 and ***p<0.001, ****p<0.0001. Primers used for gene amplification are detailed in Table 1.

### Cell cycle analysis

HaCaT cells in culture were trypsinized and immediately fixed with ice-cold 70% ethanol (Applichem GmbH, Darmstadt, Germany). Then, cells were washed three times with cold Phosphate Buffered Saline (PBS) (Biowest, Nuaillé, France) and stained for cell cycle with propidium iodide using a standard method. Briefly, cells were treated with a solution of 20 μg/ml Ribonuclease A (Sigma-Aldrich, St Louis, MO, USA) and 40 μg/ml propidium iodide (Sigma-Aldrich, St Louis, MO, USA) in PBS. Then, cells were analysed by flow cytometry using a FACS Calibur 1 (Beckton Dickinson, Franklin Lakes, NJ, USA).

### Antibodies

The following commercial antibodies were used: anti-phospho-ERK1/2, anti-Growth Factor Receptor-Bound Protein 2 (Grb2), anti-phospho-p38 kinase, anti-phospho-JNK1/2, anti-phospho-MKK3/6, anti-phospho-Smad2, anti-phospho-Smad3 and anti-phospho-c-Jun (all Cell Signaling Technology, Danvers, MA, USA); anti-CDKN1A (p21), anti-CDKN2B (p15), anti-Smad4, anti-Zonula occludens (ZO)-1 and anti-c-Jun (all Santa Cruz Biotechnology, Heidelberg, Germany); anti-Smad2/3 (BD Transduction Laboratories, Beckton Dickinson, Franklin Lakes, NJ, USA); and anti-β-Actin (Sigma-Aldrich, St Louis, MO, USA). Secondary antibodies: HRP rat anti-mouse IgG1 (BD Pharmingen, Beckton Dickinson, Franklin Lakes, NJ, USA), anti-rabbit IgG horseradish (Ge Healthcare), Alexa fluor 594-labelled phalloidin (Molecular Probes, Thermo Fisher Scientific, Waltham, MA USA), which was used to reveal actin filaments, Alexa fluor 488-labeled goat anti-mouse IgG and Alexa fluor 488-labeled goat anti-rabbit IgG (Molecular Probes, Thermo Fisher Scientific, Waltham, MA USA).

### In vitro wound healing assay

The nonmalignant mink lung epithelial cells Mv1Lu [33–35] or HaCaT cells [32]were used to evaluate cell migration. Both Mv1Lu and HaCaT cells were grown on 12-well culture plates. Cells were grown until they reached 100% confluence, then cells were serum starved for 24 h before the experiment to reduce cell proliferation. When indicated, immediately before wounding, cells were treated for 3 h with 10 μg/ml of Mitomycin C (MMC) (Sigma-Aldrich, St Louis, MO, USA) to prevent cell proliferation. Afterwards, cells were washed with medium twice and appropriate medium without serum was added. Then, the fully confluent sheet of cells was scratched with a 200 μl sterile tip. Subsequently, cultures were treated with EGF (10ng/ml) (Sigma-Aldrich, St Louis, MO, USA) or AM portions alone or in combination with different inhibitors. Pictures were taken, before and several hours (indicated in each experiment) after treatment, with a Moticam camera 2300 3.0 M Pixel USB 2.0 coupled with an optical microscope (Motic Optic AE31 from Motic Spain, Barcelona, Spain).

### Immunocytochemistry of marker expression in wound healing assay

For the immunofluorescence of wound healing assay on cultured cells, both HaCaT and Mv1Lu cells were plated on round cover glass until they reached 100% confluence, then cells were serum starved for 24 h before the experiment to induce cell cycle arrest. When indicated, immediately before wounding, cells were treated for 3 h with 10 μg/ml MMC (Sigma-Aldrich, St Louis, MO, USA) to decrease cell proliferation. Afterwards, cells were washed with medium twice and appropriate medium without serum was added. Then, the epithelium was wounded with a razor blade, which was immediately dragged to create a space large enough to allow migration for at least 24 h. Subsequently; cultures were treated with either EGF (10ng/ml)(Sigma-Aldrich, St Louis, MO, USA) or AM portions alone or in combination with different inhibitors. After a period of time, conveniently indicated in the figure legend for each experiment, cells were fixed for 10 min with 4% formaldehyde (Applichem GmbH, Darmstadt, Germany) in PBS (Biowest, Nuaillé, France) at room temperature. Subsequently, cells were permeabilised with 0.3% Triton X-100 (Sigma-Aldrich, St Louis, MO, USA) in PBS (Biowest, Nuaillé, France) for 15 min and then incubated with blocking buffer [0.3% Bovine Serum Albumin (BSA) (Santa Cruz Biotechnology, Heidelberg, Germany), 10% FBS (Thermo Fisher Scientific, Waltham, MA, USA), 0.1% Triton X-100 (Sigma-Aldrich, St Louis, MO, USA) in PBS (Biowest, Nuaillé, France)] supplemented with 5% skimmed milk (Beckton Dickinson, Franklin Lakes, NJ, USA), at room temperature for half an hour. Subsequently, samples were incubated for 2 h with an appropriate primary antibody diluted in blocking buffer, and then washed three times with 0.1% Triton X-100 (Sigma-Aldrich, St Louis, MO, USA) in PBS (Biowest, Nuaillé, France) for 10 min. After staining with the primary antibody, samples were stained with the appropriate fluorescent-labelled secondary antibodies together with Alexa fluor594-labelled phalloidin (Molecular Probes, Thermo Fisher Scientific, Waltham, MA USA) and Hoechst 33258 (Fluka, Biochemika, Sigma-Aldrich, St Louis, MO, USA) for 1 h at room temperature. Finally, samples were examined and representative images were taken with a confocal microscope (LSM 510 META from ZEISS, Jena, Germany).

### AM processing, preparation and wound healing application

Term placenta from healthy donor mothers was obtained from uncomplicated caesarean section. The foetal membranes were washed in Physiological Saline Solution (PSS) (B.Braun, Barcelona, Spain) supplemented with 50 μg/ml Amphotericin (Bristol-MyersSquibb, Madrid, Spain), 48 μg/ml Clotrymazol (Almirall-Prodesfarma, Barcelona, Spain), 50 μg/ml Tobramycin (Laboratorios Normon, Madrid, Spain) and 50 μg/ml Vancomycin (Laboratorios Hospira, Madrid, Spain) and rapidly transferred to the laboratory in sterile conditions. Under a laminar flow cabinet, the amnion was mechanically peeled from the chorion, washed three to four times with 200 ml of PBS (Biowest, Nuaillé, France) and flattened onto sterile nitrocellulose paper (Pierce, Thermo Fisher Scientific, Waltham, MA USA) with the basement membrane surface up. For wound healing application we followed the procedure that has been extensively described [3]. Briefly, processed pieces of AM were taken to the surgical room and applied onto the wound of the selected patient with large-surface and deep traumatic wounds with patent retard in epithelialization. The follow up of the wound was carried out as described elsewhere [3]. To evaluate the effect of AM in wound epithelialisation, biopsies were obtained from newly formed skin in patients. The samples were prepared following standard procedures for paraffin-embedded sections that were stained with haematoxylin and eosin and evaluated under the microscope by a pathologist. Immunohistochemistry on paraffin-embedded sections was performed following standard procedures. Briefly, sections were incubated with blocking buffer [3% BSA (Santa Cruz Biotechnology, Heidelberg, Germany), 0.1% Tween-20 (Sigma-Aldrich, St Louis, MO, USA) in PBS (Biowest, Nuaillé, France)] at room temperature for 1 h. Subsequently, the sections were incubated with the primary antibodies in blocking buffer at room temperature for 2 h, washed three times in PBS (Biowest, Nuaillé, France)/0.1% Tween-20 (Sigma-Aldrich, St Louis, MO, USA) for 10 min, and then incubated at room temperature for 1 h with secondary antibodies and Bisbenzimide (Hoestch-33258) 1.2 μg/ml (Fluka, Sigma-Aldrich, St Louis, MO, USA). After several washings with PBS, the sections were mounted and examined by confocal microscopy (LSM 510 META from ZEISS, Jena, Germany).

## Results

### Amniotic membrane attenuated the TGFß-induced phosphorylation of Smad2 and 3

**Fig 1:**
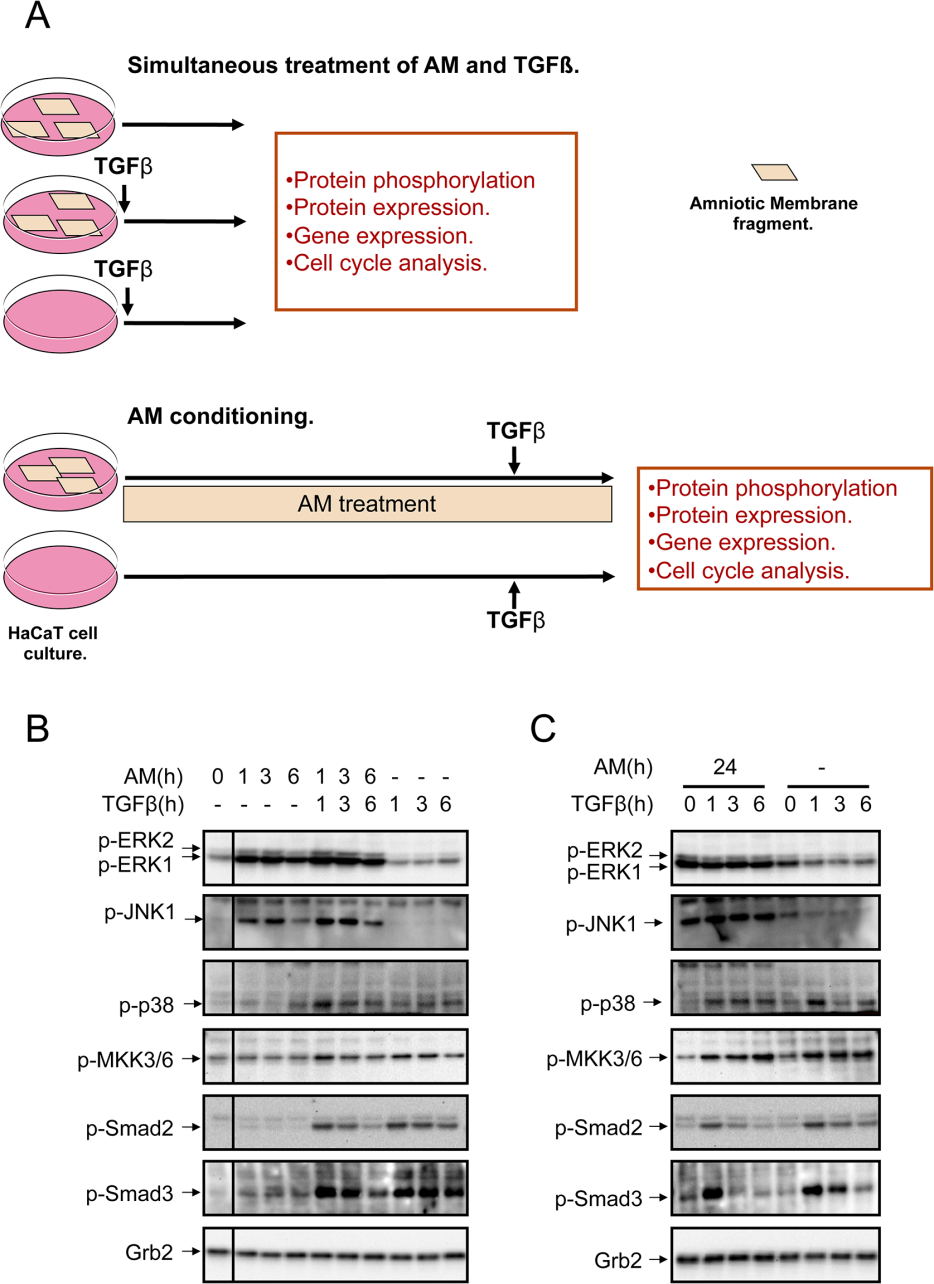
AM attenuated TGFß-induced phosphorylation of Smad2 and 3 in HaCaT cells. (A) Representative scheme of the experimental approach followed for the treatment of HaCaT cells with AM, TGFß or both together. Alternatively, HaCaT cells were conditioned for 24 h treatment with AM and then treated with TGFß. In both cases, after treatment several signaling pathways were studied. (B), HaCaT cells were stimulated with AM, TGFß or both simultaneously for the indicated times. (C), HaCaT cells were stimulated for 24 h with AM and then treated with TGFß for the indicated times, non treated cells were used as the control. In (B) and (C), indicated proteins were detected by Western blot. Grb2 was used as a loading control. This experiment was repeated at least three times. A representative result is shown.

We have previously described the effect of AM on wounds. The application of AM improved the epithelialisation of wounds allowing for their spontaneous closure without further intervention [3]. In order to study the possible effects of AM on keratinocytes we used HaCaT cells that have been widely used as a model for keratinocyte behaviour. HaCaT cells largely retain their capacity to reconstitute a well-structured epidermis after transplantation in vivo [32]. Previously, we have shown that AM induced the phosphorylation of ERK as well as JNK1 but not of p38 or MKK3/6 [4]. The experimental approach is detailed in Fig 1A. HaCaT cells in culture were stimulated with AM at several time points, and to study the implications of AM treatment for TGFß responses, cells were also co-stimulated with TGFß or only stimulated with TGFß as a control at the same time points. The stimulation of HaCaT cells with AM induced a strong and robust phosphorylation of ERK (Fig 1B) and the phosphorylation of JNK1, as previously seen [3]. However, when cells were co-stimulated with AM and TGFß, JNK1 and ERK phosphorylation were further increased (Fig 1B). Also, the presence of AM seemed to enhance the TGFß phosphorylation of p38 and MKK3/6 (Fig 1B) although little stimulation was seen in cells stimulated with AM alone (Fig 1B). To see whether AM could modify the response of HaCaT cells to TGFß, we cultivated cells for 24 h in the presence of AM before being stimulated with TGFß (See Fig 1A). In such a case, a strong stimulation of ERK1 and 2 was sustained for 24 h and beyond and was neither enhanced nor prevented by TGFß (Fig 1C). When we looked into the JNK1 phosphorylation, the stimulation with AM sustained its effect for 24 h and TGFß did not stimulate it further. We studied the effect of AM on the phosphorylation of receptor regulated Smads. Cells grown in the presence of AM attenuated the phosphorylation of Smad2 upon TGFß stimulation when compared to control cells. The phosphorylation of Smad2, in response to TGFß, was attenuated by the presence of AM either when cells were stimulated with TGFß simultaneously to AM or when AM treatment was followed sequentially by TGFß stimulation (Fig 1B and 1C). A similar result was obtained for the TGFß phosphorylation of Smad3 (Fig 1B), which was even more attenuated when cells were incubated with AM for 24 h (Fig 1C). Taken together, these data indicate that the presence of AM and TGFß in HaCaT cells modified the phosphorylation of JNK1 kinase that is produced by AM [3]. Additionally, the presence of AM attenuates the TGFß-induced phosphorylation of Smad2 and Smad3for HaCaT cells.

### AM caused an attenuation of TGFß-induced expression of CDKN1A (p21) and CDKN2B (p15)

**Fig 2:**
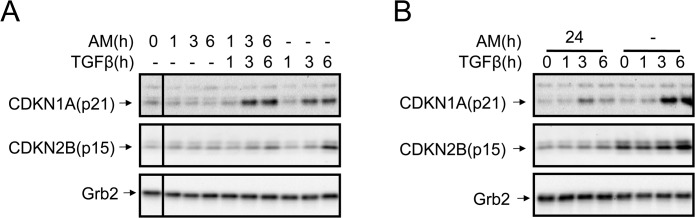
AM treatment of HaCaT cells attenuated the expression of CDKN1A (p21) and CDKN2B (p15) proteins. (A), HaCaT cells were stimulated with AM, TGFß or both simultaneously for the indicated times. (B), HaCaT cells were stimulated for 24 h with AM and then treated with TGFß for the indicated times, as a control non treated cells were used. Indicated proteins were detected by Western blot. Grb2 was used as a loading control. This experiment was performed at least three times. A representative result is shown.

Because AM attenuates TGFß-induced Smad2 and 3 phosphorylation in HaCaT, we investigated whether this may have a consequence for the expression of proteins related to cell cycle regulation. Thus, we measured the expression of two TGFß-regulated cell cycle key proteins, CDKN1A (p21) and CDKN2B (p15), in response to TGFß either in the presence or absence of AM. Simultaneous stimulation of HaCaT cells with AM and TGFß only had an effect on TGFß–upregulation of CDKN2B (p15) (Fig 2A). In contrast, when cells were AM conditioned for 24 h, both CDKN1A (p21) and CDKN2B (p15) TGFß-induced expression was inhibited (Fig 2B). This data indicates that the effect of AM on TGFß/Smad signalling has an effect on the expression of the TGFß regulated proteins CDKN1A (p21) and CDKN2B (p15).

### AM regulated the expression of several TGFß-dependent genes

To know to what extent AM treatment affects the TGFß gene responses, we studied the expression of a panel of genes representing different types of response to TGFß stimulation. We chose genes with either a rapid response to TGFß, with maximal expression 1 h after TGFß treatment, or with a slow response to TGFß [36], genes whose response was increasing at least 6 h after TGFß induction. Among all these genes, there were genes whose response was either dependent or independent of Smad4 [36]. We studied gene expression in both experimental sets: cells that have been preconditioned with AM for 24 h and then stimulated with TGFß or cells in which the stimulation with AM and TGFß was simultaneous (See Fig 1A).

**Fig 3:**
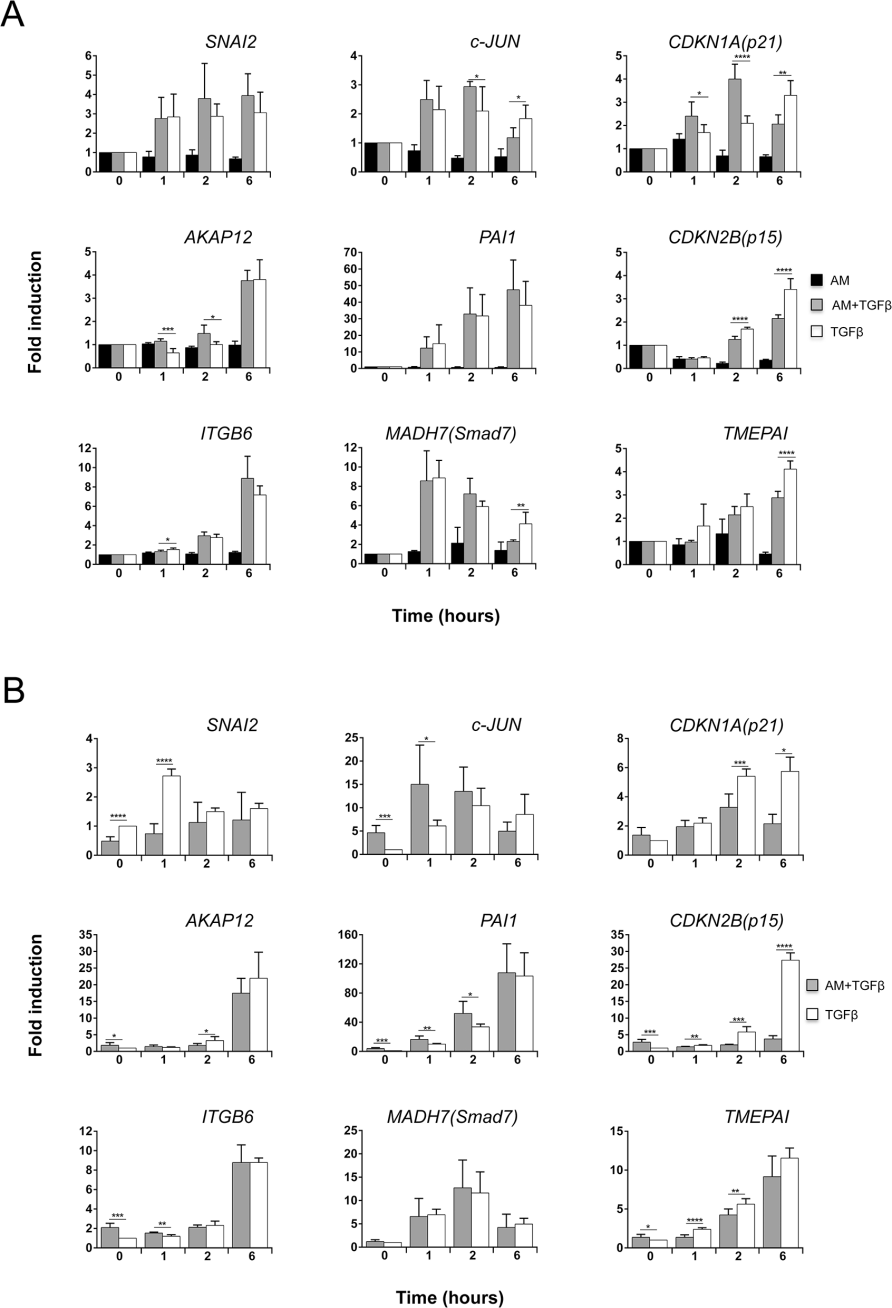
AM modified the genetic response induced by TGFß in HaCaT cells. Several TGFß inducible genes were measured in HaCaT cells stimulated with TGFß or TGFß and AM. (A), isolated RNA from HaCaT stimulated with AM, TGFß, or both was analyzed by qPCR, represented as a ratio to *GAPDH* and represented as fold change of the untreated control sample. (B), isolated RNA from HaCaT stimulated for 24 h with AM and TGFß for the indicated times, or only with TGFß was analysed by qPCR, represented as a ratio to *GAPDH* and represented as fold change of the untreated control sample. The asterisks denote statistically significant differences between the treatments according Student’s *t*-test. *p<0.05, **p<0.005 and ***p<0.001, ****p<0.0001.

AM produced variations in the TGFß-induced expression of some genes. Among these, *CDKN1A (p21)* was highly induced or *SMAD7* and *TMEPAI* slightly decreased at 6 h by TGFß in the presence of AM (Fig 3A). Interestingly, the expression of *CDKN2B (p15)* in response to TGFß was decreased when co-cultured with AM (Fig 3A). None of the other examined genes experienced significant changes when stimulated with TGFß in the presence or absence of AM. Interestingly, when cells were AM pre-treated for 24 h gene responses were different. AM pre-treatment affected the TGFß response of *SNAI2* and *CDKN1A (p21)* (Fig 3B), and strikingly the TGFß expression of *CDKN2B (p15)* was severely prevented by the presence of AM (Fig 3B). Additionally, we observed a rise in *c-JUN* in cells treated with AM for 24 h. Moreover, treatment with AM further enhances the expression of *c-JUN* in response to TGFß (Fig 3B).

Altogether, this data point to the fact that AM opposes the upregulating capacity of TGFß for at least *CDKN1A (p21)* and *CDKN2B (p15)* while acting synergistically on the TGFß expression of *c-JUN*.

### The effect of AM on human primary keratinocytes

**Fig 4:**
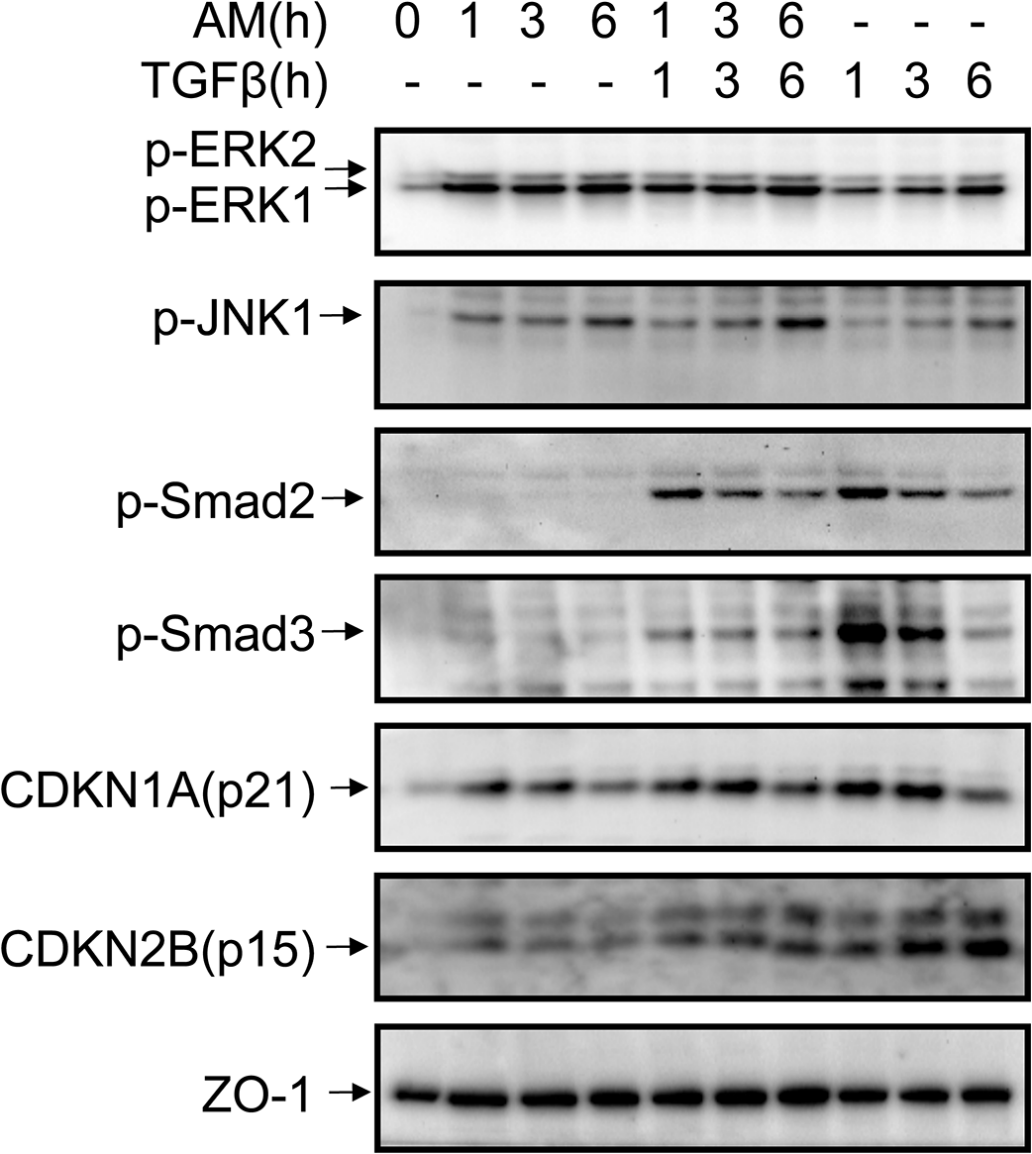
AM treatment induced the activation of several signalling pathways in human primary keratinocytes. A similar experimental approach was followed to the one shown in Fig 1A. Human primary keratinocytes were stimulated with AM, TGFß or both simultaneously for the indicated times, non treated cells were used as the control. Indicated proteins were detected by Western blot. ZO-1 was used as a loading control. This experiment was repeated at least three times. A representative result is shown.

**Fig 5:**
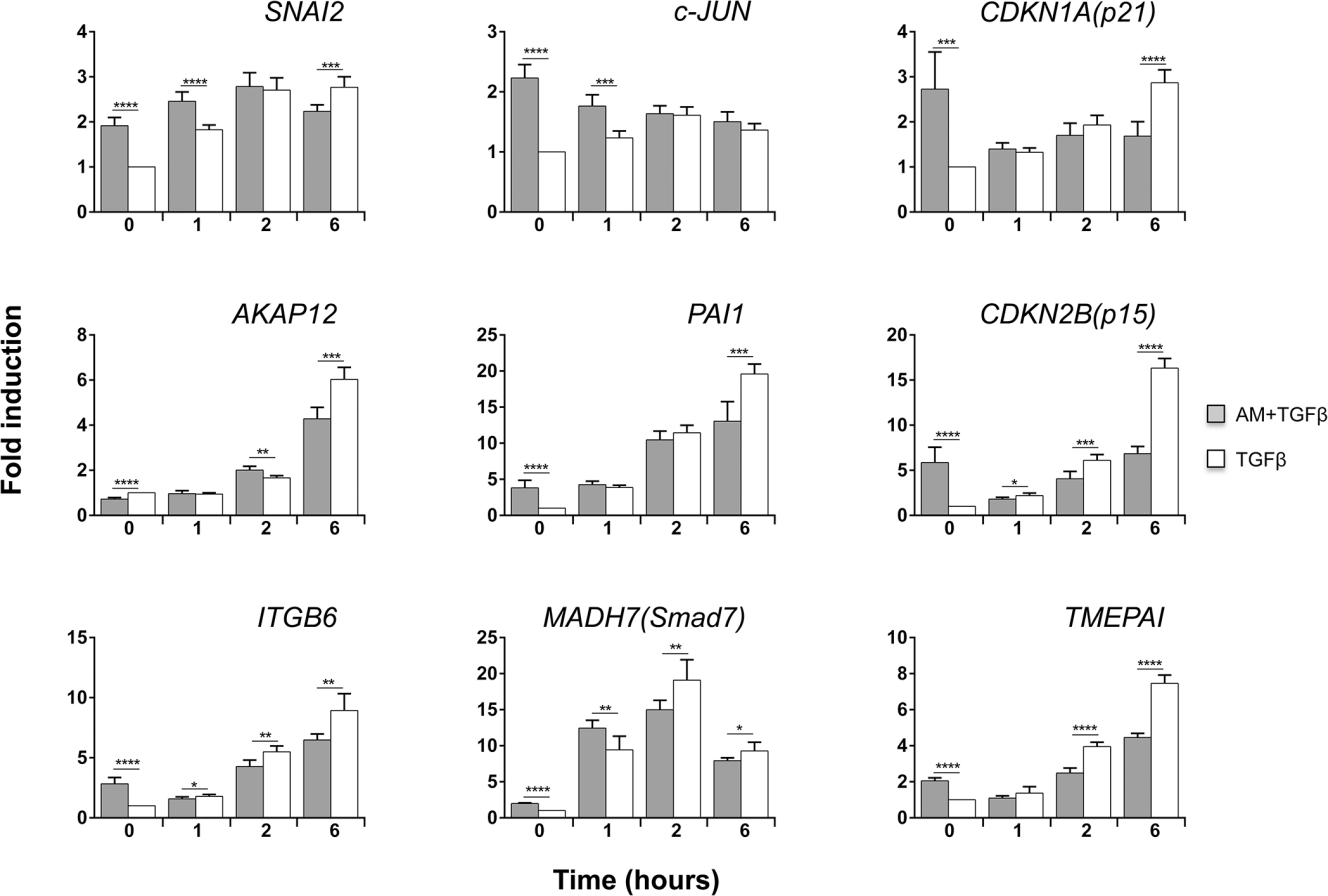
AM modified the genetic response induced by TGFß in human primary keratinocytes. Several TGFß inducible genes were measured in human primary keratinocytes in response to TGFß or in response to the combined treatment of TGFß and AM. Isolated RNA from primary keratinocytes stimulated for 24 h with AM and TGFß for the indicated times, or only with TGFß was analysed by qPCR, represented as a ratio to *GAPDH* and represented as a fold change of the untreated control sample. The asterisks denote statistically significant differences between the treatments according Student’s *t*-test. *p<0.05, **p<0.005 and ***p<0.001, ****p<0.0001.

The human keratinocyte cell line HaCaT essentially expresses all epidermal differentiation markers but exhibits deficiencies in tissue organization in surface transplants in nude mice or in organotypic co-cultures with fibroblasts [37]. Therefore, we decided to apply our experimental approach to human primary keratinocytes. Human primary keratinocytes induced the phosphorylation of Smad2 and Smad3 for at least 6 h upon TGFß stimulation, in a similar way to HaCaT and other known epithelial cells (**S1 Fig**). The cells also exhibit normal levels of Smad2, Smad3 and Smad4, anticipating a complete and functional TGFß signalling pathway [38, 39]. Additionally, stimulation with TGFß induced the expression of proteins required for TGFß cell cycle control such as CDKN1A (p21) and CDKN2B (p15) (**S1 Fig**). We used the same experimental approach as in Fig 1A for studying the effect of AM upon TGFß signalling in primary keratinocytes. Both ERK 1 and 2 and JNK1 phosphorylation were increased by the presence of AM, and the presence of TGFß slightly increased the phosphorylation of JNK1 (Fig 4). The consequences of AM co-culturing over TGFß phosphorylation of Smads were similar to those found in HaCaT cells: AM caused an attenuation of TGFß signalling that was especially evident for Smad3 phosphorylation (Fig 4). In parallel, TGFß-induced expression of proteins CDKN1A (p21) and CDKN2B (p15) was attenuated upon AM treatment, and similarly to HaCaT cells, the effect was more accused on the expression of CDKN2B (p15) (Fig 4). To extend the evaluation of the effect of AM on human primary keratinocytes, we studied the AM effect on the expression of the same TGFß regulated genes as in HaCaT. AM (24-h treatment) negatively affected the TGFß-induced gene expression program in human primary keratinocytes for cell cycle regulator genes *CDKN1A (p21)* and *CDKN2B (p15)*, while inducing a positive expression for *c-JUN* (Fig 5). Moreover, AM treatment for 24 h induced the expression of *ITGB6*, *TMEPAI*, *SNAI2* and *PAI1* when compared to the control sample, and did not prevent further induction by TGFß (Fig 5). Essentially, human primary keratinocytes exhibit a protein and gene response that is consistent with the effect of AM on wound healing.

### AM treatment of HaCaT cells attenuated the cell cycle arrest induced by TGFß

**Fig 6:**
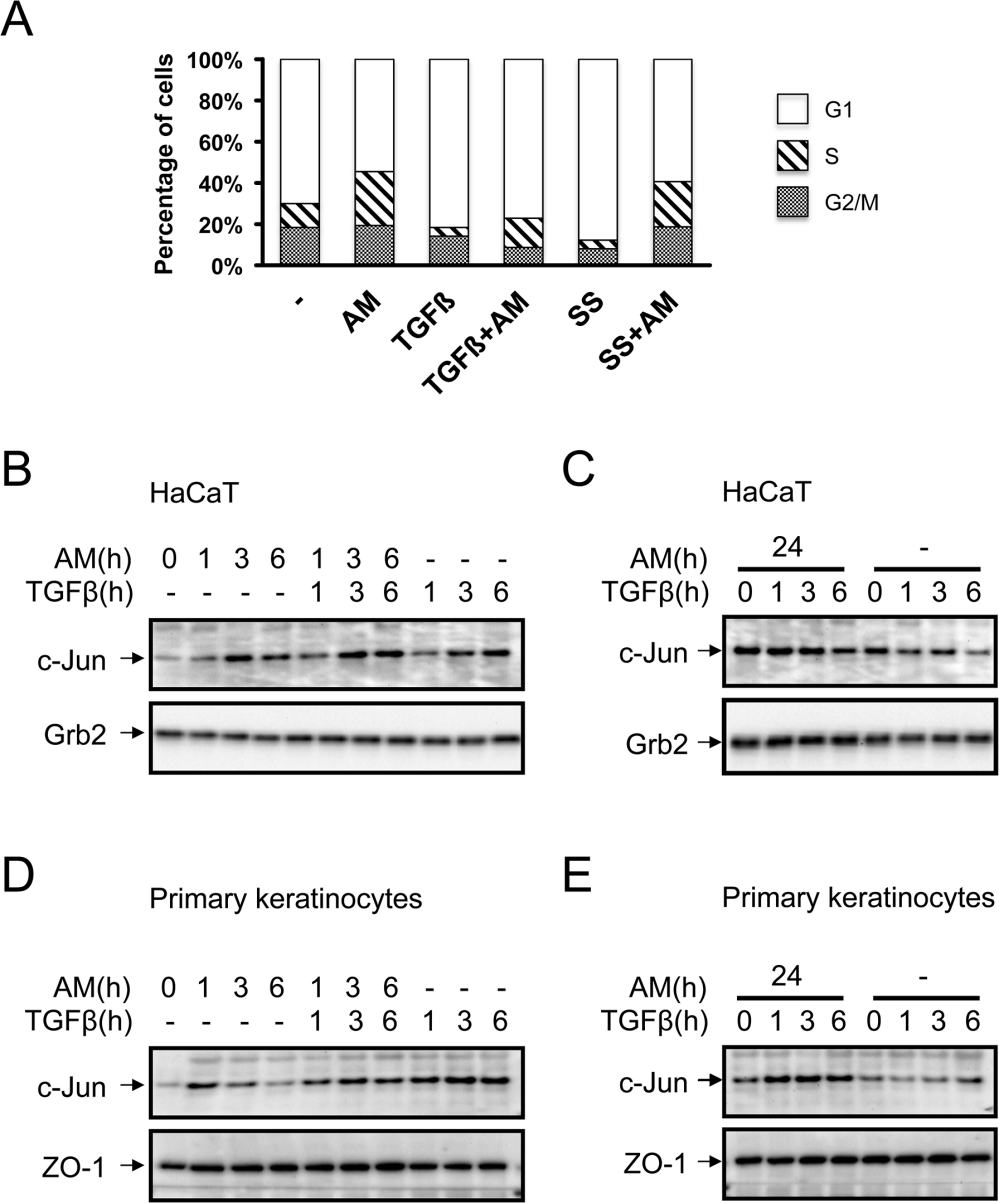
AM attenuated cell cycle proliferation arrest of TGFß on HaCaT cells and induced the expression of c-Jun protein in HaCaT and in human primary keratinocytes. Treatment of HaCaT cells with AM attenuates TGFß-induced cell cycle arrest in G1. (A), Cell cycle analysis of HaCaT cells in different conditions, treatment with AM, combined with serum starvation (SS) or TGFß is indicated. The histogram shows cells at G0/G1, S or G2/M stage respectively. AM induced the expression of c-Jun in HaCaT and human primary keratinocytes in clear synergy with TGFß. (B), HaCaT cells or, (D), human primary keratinocytes were stimulated with AM, TGFß or both simultaneously for the indicated times. Additionally, (C), HaCaT cells, or, (E), human primary keratinocytes, were stimulated for 24 h with AM and then treated with TGFß for the indicated times, as a control non treated cells were used. Indicated proteins were detected by Western blot. Grb2 or Zo-1 were used as loading controls where indicated. This experiment was performed at least three times. A representative result is shown.

HaCaT cells respond to TGFß exhibiting a robust cell cycle arrest in G1 [40]. We have seen a powerful effect of AM on the expression of two key cell cycle control proteins, CDKN1A (p21) and CDKN2B (p15), whose expression is regulated by TGFß. To see the effect of AM on TGFß cell cycle regulation, we treated HaCaT cells with TGFß for 48 h in the presence or absence of AM, and serum starvation treatment was used as a control. While TGFß caused a clear cell cycle arrest, treatment with AM was able to relieve the arrest imposed by TGFß (Fig 6A). This data supports the idea that the attenuation of TGFß-upregulation of CDKN1A (p21) and CDKN2B (p15), in cells treated with AM, had a consequence on TGFß cell cycle control. Therefore, the stimulation of HaCaT cells with AM had a consequence on CDKN1A (p21) and CDKN2B (p15) expression that could explain the inability of TGFß to elicit the arrest of cell cycle in cells treated with AM.

### AM induced the expression of c-Jun in HaCaT cells and primary keratinocytes

Our results showed that HaCaT cells stimulated with AM increase the phosphorylation of ERK1 and 2, and AM also stimulates JNK1 phosphorylation and both have long term effects (see Fig 1B and 1C). Moreover, our results showed AM stimulation of *c-JUN* gene expression that is synergistic with the treatment with TGFß. In order to understand the molecular mechanisms underlying the improvement of AM on wound healing, we studied c-Jun protein expression in HaCaT cells stimulated with AM and compared it to cells stimulated only with TGFß or AM/TGFß. Separately, AM and TGFß upregulated the expression of c-Jun, however, together they synergistically increased the expression of it (Fig 6B). In contrast, when compared to the control sample, HaCaT cells cultivated with AM for 24 h showed a high expression of c-Jun that was not further increased upon TGFß stimulation (Fig 6C). This is consistent with the data observed at the RNA level. In primary keratinocytes however, we found that TGFß potentiated the effect of AM although the effect of both together was weaker than TGFß alone (Fig 6D). Strikingly, when primary keratinocytes were conditioned with AM for 24 h, a higher TGFß induction of c-Jun was observed when compared to control cells (Fig 6E). Altogether, this data indicates that both AM and TGFß synergistically upregulate the expression of c-Jun.

### AM induced the expression of c-Jun at the edge of a scratch wound assay on Mv1Lu cells

Cell migration is one of the main driving processes for wound healing. In keratinocytes, c-Jun is a key component of the migration mechanism. Phosphorylation of p38 has also been associated with cell migration [41]. To see whether AM may have an effect on cell motility; we assayed AM on Mv1Lu cells [42]. Mv1Lu cells are widely used to test the effect of different stimuli on cell migration [33–35]. As we have shown above, AM induces the phosphorylation of ERK1 and 2 in both HaCaT cells and primary keratinocytes (see Figs 1B, 1C and 4). Strikingly, AM induced the migration of Mv1Lu cells, and as a control of positive cell migration we used EGF (**S2A Fig**). In order to ascertain the molecular mechanisms involved in AM-induced cell migration, we used several well-characterized inhibitors. The migration of cells, in response to AM, was prevented by the JNK and MEK inhibitors, SP600125 and PD98059 respectively, but not by the p38 kinase inhibitor, SB203580 (**S2A Fig**). The stimulation of Mv1Lu cells with AM induced the phosphorylation of ERK1 and 2, and such phosphorylation was prevented by the usage of the MEK inhibitor, U0126, but not by the JNK inhibitor SP600125 (**S2B Fig**). The AM-induced c-Jun expression was partially prevented by both the JNK and MEK inhibitors (**S2B Fig**). AM also induced the phosphorylation of c-Jun, that was prevented by the usage of both the JNK and the MEK inhibitors (**S2B Fig**). These results are consistent with the cell migration results and suggest that the effect of AM on cell migration may be, at least partly, attributed to the activation of the JNK and MEK kinase pathways. Both of them cause the phosphorylation of c-Jun and are partially involved in the transcription activation of *c-JUN*. However, p38 kinase is not involved in AM cell migration.

To evaluate the net contribution of cell migration to the AM wound healing effect, cells were treated with Mitomycin C (MMC) prior to the wound healing experiment. MMC treatment prevented cell proliferation (data not shown). In this case, the presence of AM produced a very positive effect on cell migration that was prevented by the JNK and MEK inhibitors, SP600125 and U0126 respectively, but not by the p38 kinase inhibitor, SB203580 (**S3A Fig**). Similar results were found when AM was tested in MDA-MB-231 cells (Data not shown), a human breast cell line also used in wound healing experiments [43–45]. All these results suggest a net contribution of AM to cell migration, irrespective of cell proliferation, that is dependent on JNK and MEK kinases but not on p38 kinase.

We next tested the expression of c-Jun at the wound border of the wound healing assay. AM clearly induced the expression of c-Jun at the edge of the wound healing scratch assay while at a certain distance from the border, where the cells formed a tight epithelium and the cells exhibited a much lower expression of c-Jun (**S2C Fig**). Similarly, the stimulation with EGF caused the cells to up regulate c-Jun expression, although the appearance of cells was slightly less compact and sparser compared to AM treated cells (**S2C Fig**). The treatment with the p38 kinase inhibitor, SB203580, neither affected migration induced by AM nor the induction of c-Jun at the leading edge of the scratch wound assay (**S2A and S2C Fig**). However, neither the JNK nor the MEK inhibitors, SP600125 or U0126 respectively, prevented the increase in the expression of c-Jun at the wound healing edge in response to AM (**S2C Fig**). These data are fully consistent with the previous data of migration and protein expression. Migrating Mv1Lu cells, even in the presence of MMC, overexpressed c-Jun at the wound edge upon treatment with AM (**S3B Fig**), and that overexpression was abolished by the presence of the JNK and MEK inhibitors. All these data suggest that AM induced the expression of c-Jun at the wound edge irrespectively of cell proliferation, and suggest that AM enrols c-Jun, in all cases, to stimulate cell migration.

### AM induced the expression of c-Jun at the edge of a scratch wound healing assay on HaCaT cells

**Fig 7:**
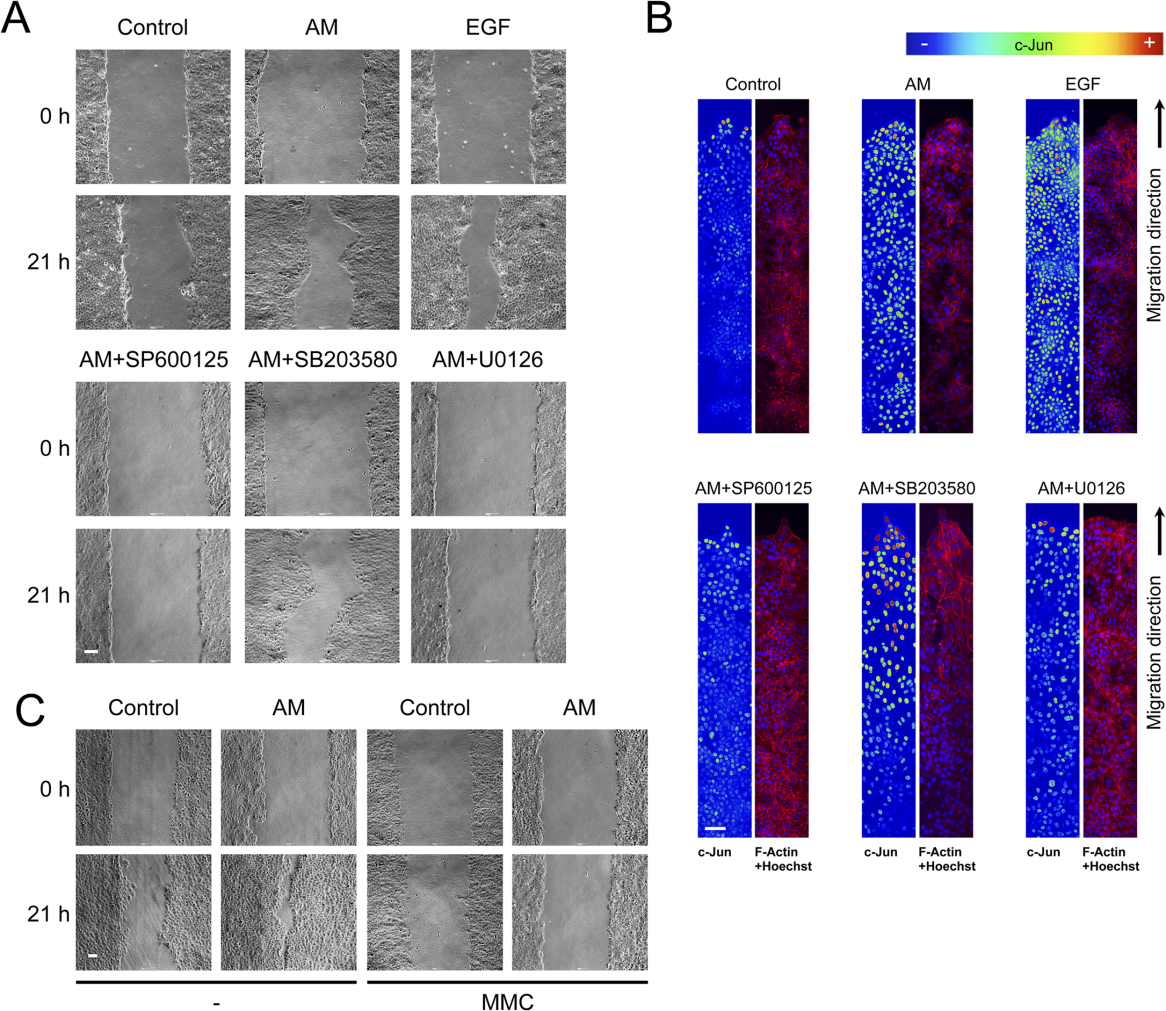
In HaCaT cells, AM induced motility and the expression of c-Jun at the migratory front. Wound healing scratch assay was performed in HaCaT cells in the presence of AM, EGF or combinations of AM with different inhibitors. (A), cells forming a confluent epithelium were wounded and immediately treated as indicated for 21 h. Representative pictures were taken at the beginning of the treatment and 21 h later. (B), treatment of HaCaT cells with AM caused the cells to express c-Jun at the migratory front. Wound healing scratch assay was treated with AM, EGF or combinations of AM with different inhibitors. Cells were wounded and treated for 24 h, afterwards cells were fixed and immunostained for c-Jun. Images of c-Jun fluorescence were converted into pseudo-colour to show the intensity of c-Jun staining. Colour rainbow scale represents fluorescence intensity for c-Jun. Co-staining with phalloidin and Hoechst-33258 was used to show cells structure and nuclei, respectively. Images were taken by confocal microscopy using a Zeiss 510 LSM confocal microscope. This experiment was repeated at least three times. A representative result is shown. (C), HaCaT cells forming a confluent epithelium were treated with Mitomycin C, wounded and immediately treated for 21 h as indicated. Results were compared to non-treated cells. Representative pictures were taken at the beginning of the treatment and 21 h later. Scale Bars 100 μm.

We wanted to assay the effect of AM on keratinocytes. AM induced the migration of HaCaT cells (Fig 7A). In a similar way to Mv1Lu cells, AM-induced migration was prevented by the JNK and MEK inhibitors, SP600125 and U0126 respectively, but not by the p38 kinase inhibitor, SB203580 (Fig 7A). These results are consistent with the cell migration results and suggest that the effect of AM on cell migration may be, at least, attributed to the activation of the JNK and MEK kinase pathways. We next tested the expression of c-Jun at the wound border of the HaCaT cell wound healing assay. Here, AM clearly induced the expression of c-Jun at the edge of the wound healing scratch assay and continued with a slightly lower expression to where cells formed a tighter epithelium (Fig 7B). Similarly, the stimulation with EGF caused the cells to upregulate c-Jun expression (Fig 7B). The treatment with the p38 kinase inhibitor, SB203580, did not affect AM-induced migration and even increased further the expression of c-Jun at the wound edge. Consistent with migration data, the JNK and MEK inhibitors, SP600125 and U0126 respectively, show a lower level of c-Jun at the wound border or the inner tight epithelium that were close to the levels for the control (Fig 7B). In contrast to Mv1Lu cells, where the migration of cells fulfils the need for epithelialisation in the scratch assay, HaCaT cells required proliferation for cell migration to take place in the wound-healing assay. Indeed, in HaCaT cells, the presence of MMC prevented healing of the scratch assay (Fig 7C).

**Fig 8:**
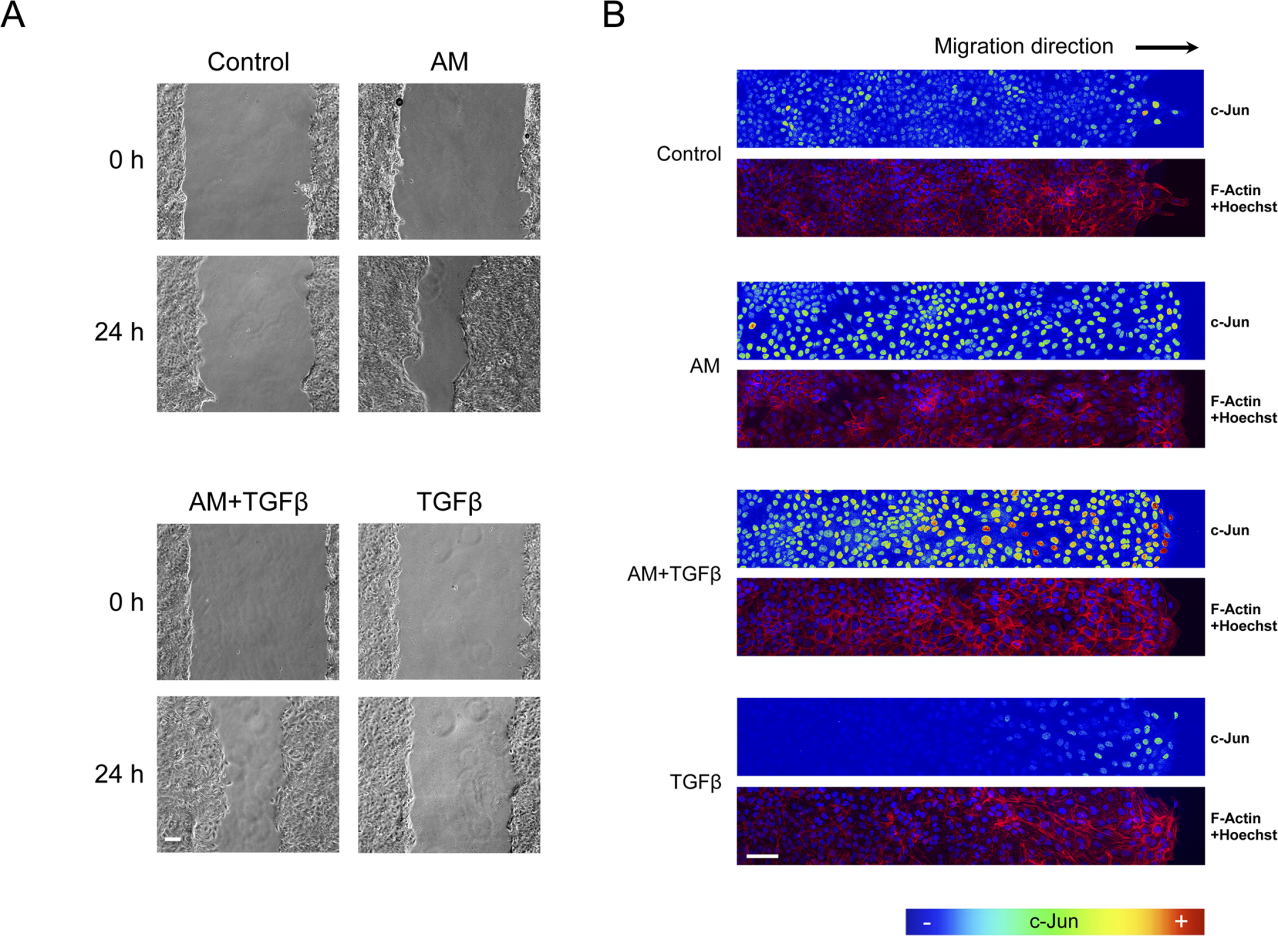
AM induced c-Jun expression is enhanced in the presence of TGFß. Wound healing scratch assay was performed in HaCaT cells in the presence of AM, TGFß or a combination of both. (A), cells forming a confluent epithelium were wounded and immediately treated as indicated for 24 h. Representative pictures were taken at the beginning of the treatment and 24 h later. (B), wound healing scratch assay was treated with AM, EGF or combinations of AM with different inhibitors. Cells were wounded and treated for 24 h, afterwards cells were fixed and immunostained for c-Jun. Images of c-Jun fluorescence were converted into pseudo-colour to show the intensity of c-Jun staining. Colour rainbow scale represents fluorescence intensity for c-Jun. Co-staining with phalloidin and Hoechst-33258 was used to show the cell structure and nuclei, respectively. Images were taken by confocal microscopy using a Zeiss 510 LSM confocal microscope. This experiment was repeated at least three times. A representative result is shown. Scale Bars 100μm.

We next tested the effect of AM on the wound-healing assay and compared it to TGFß. Indeed, while in the presence of TGFß the migration of cells was very weak, the treatment with AM led to cell migration despite TGFß (Fig 8A). We have shown that AM overruled the cell cycle arrest effect of TGFß on HaCaT cells (See Fig 6A). Consistently, in the wound healing scratch assay, the presence of AM increased the number of proliferating cells compared to the control sample, and more importantly overruled the negative effect of TGFß on cell proliferation (Data not shown). Next we assayed the expression of c-Jun at the borders of the wound healing scratch assays. While the control sample exhibited some expression of c-Jun, treatment with AM increased the expression of c-Jun very strongly, to a much further extent than in Mv1Lu cells. More importantly, while treatment with TGFß exhibited a similar c-Jun expression to the control, the simultaneous treatment of HaCaT wounds with AM and TGFß increased the expression of c-Jun very strongly, especially at the wound border (Fig 8B). As in the Mv1Lu cells, AM induced the phosphorylation of c-Jun, and the induction of phosphorylation was even stronger in the presence of both TGFß and AM (Data not shown).

All these results, in HaCaT cells, suggest a net contribution of AM to cell migration that is strongly dependent on cell proliferation and also dependent on JNK and MEK kinases but not on p38 kinase. Additionally, AM also enrols c-Jun to stimulate cell migration in synergy with TGFß, at the same time as AM counteracts the antiproliferative effects of TGFß on keratinocytes.

### AM increased the expression of c-Jun at the edge of AM treated wounds

**Fig 9:**
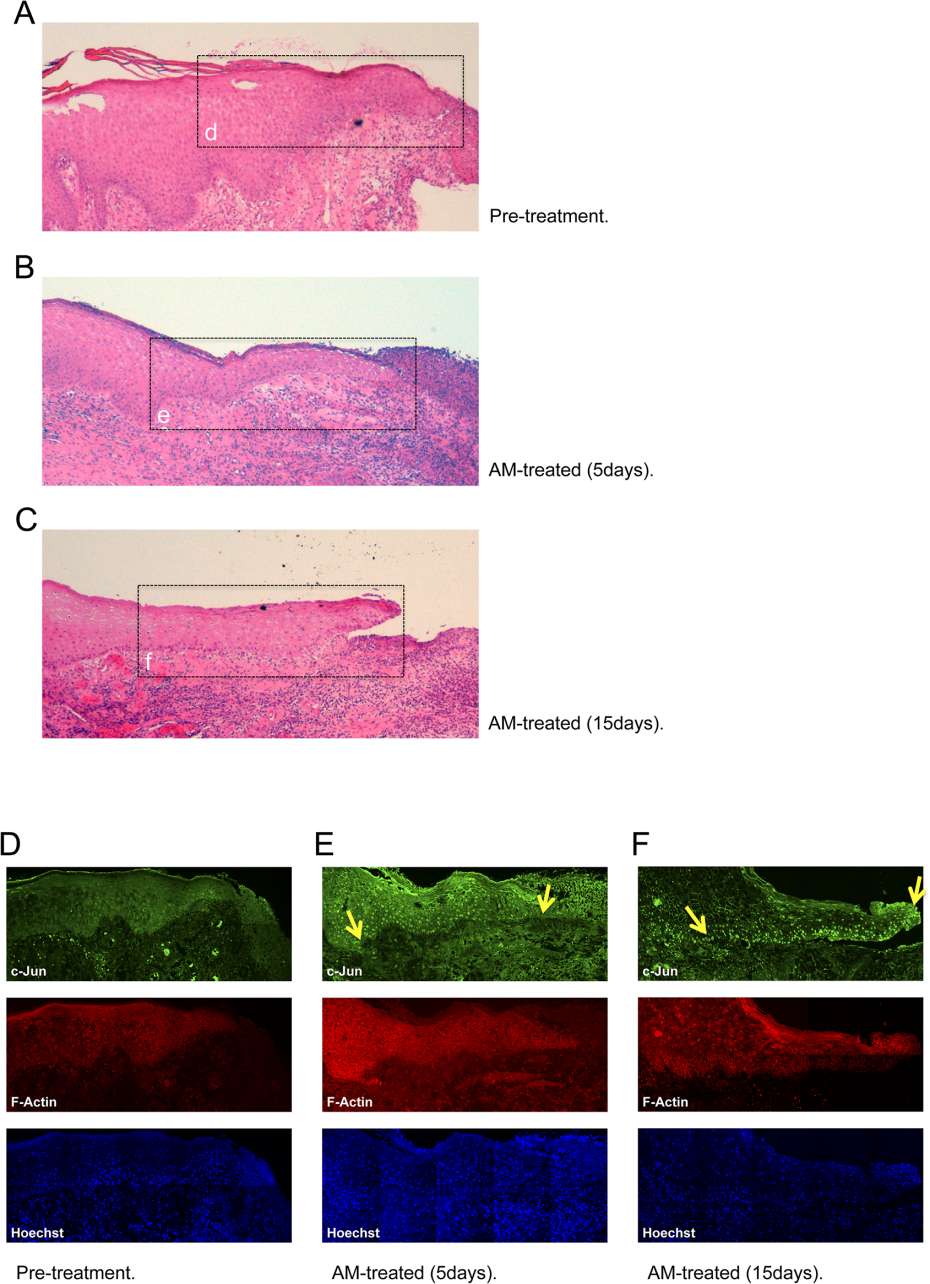
Expression of c-Jun at the epidermal leading edge. Histopathological study of AM induced epithelialisation from a patient´s wound that had been treated with AM. (A), microscopic section from wound border before AM treatment. (B), and (C), microscopic section of the wound border 5 and 15 days after AM treatment, respectively. Insets in Fig A to C show roughly the area where confocal microscopy images have been taken. (D), to (F), microscopic sections were also immune-stained against c-Jun (green) and F-Actin (red). Cell nuclei were revealed by Hoechst-33258 staining. (D), same as in A; (E), same as in (B) and (F), same as in (C). Arrows in (E) and (F) point to the epidermal leading edge. Several patients were analysed in this experiment, single patient data is shown for illustrative purposes. Images were taken by confocal microscopy using a Zeiss 510 LSM confocal microscope.

AM-induced motility depends on c-Jun expression. Also, AM induces the expression of c-Jun at the border of the scratch wound assay. Therefore, we examined the wound border of several patients that had been treated with AM for c-Jun expression. These patients had been included in a compassionate use of AM for big-wound healing. Wound border samples were taken before and after AM application. The border of the paraffin embedded section of an untreated wound of one of the patients showed a blunt end with no apparent formation of keratinocyte tongue (Fig 9A). However, 5 or 15 days after treatment with AM, the section at the wounds edge clearly showed a migrating epithelial tongue (Fig 9B and 9C), typically constituted by keratinocytes that are regenerating the epidermis [46]. Previously, our group had observed that the presence of AM correlated with the high expression of c-Jun in human skin wound border basal stratum keratinocyte, although the timely effect of the AM was not assessed [3]. In our patients, wounds that had been treated for several days to induce granulation of the dermis and underlying tissue showed no evidence of c-Jun expression at the wound border (Fig 9D). Strikingly, the presence of AM caused the basal keratinocytes of the epidermal leading edge to increase the expression of c-Jun (Fig 9E), that was kept high for several days after AM stimulation (Fig 9F).

## Discussion

Wound healing is a complex process that includes inflammation, re-epithelialisation, neoangiogenesis and tissue remodelling with the aim of restoring tissue integrity in the wounded area [47, 48]. If the normal process of wound healing is disrupted, a chronic non-healing wound can result. Chronic wounds are, by definition, wounds that have failed to progress through the normal stages of healing and therefore enter a state of pathological inflammation. As a result, the healing process is delayed, incomplete and does not proceed in a coordinated manner, subsequently resulting in poor anatomical and functional outcome [49].

We have shown that treatment of massive chronic wounds with AM induces a robust epithelialisation that finally covers the wound [3]. High levels of TGFß had been found in chronic non-healing wounds [50, 51]. The application of AM may antagonise the TGFß signalling, and to ascertain that, we have used the human keratinocyte cell model (HaCaT) to study the effect of AM on the TGFß induced cell response. AM produces many molecules, that are able to induce several signalling pathways. So far, we have detected the activation of ERK phosphorylation as well as the induction of JNK1 phosphorylation and c-Jun phosphorylation. However, the overall clinical experience with growth factors and other mediators to accelerate wound healing has been discouraging. This is not surprising, considering that wound repair is the result of a complex set of interactions among soluble cytokines, formed blood elements, the extracellular matrix and cells [47]. The mixture of different growth factors in a precise combination has been suggested to be more effective for the promotion of wound healing [47]; moreover, neonatal epidermal cells release growth factors that stimulate other epidermal cells [47]. Although AM produces several cytokines and growth factors, we do not know which of these may be involved in the effect of AM on wound epithelialisation. Indeed, knowing the molecular factors involved on the effect of AM on chronic wounds epithelialization would very important to decipher the complete molecular code that AM triggers in keratinocytes. This is a research task that we want to undertake in the near future. Despite this, one of the more striking features that we have observed when AM was used on HaCaT cells was its ability to attenuate the TGFß-induced phosphorylation of Smad2 and Smad3. The strength and duration of TGFß signalling, expressed as continuous phosphorylation of Smads, are required to achieve proper cell responses to TGFß [38]. There has been a deep interest in the down-regulation of TGFß as a way to improve wound healing. For instance, treatment of human keratinocytes with TGFß antisense oligonucleotides may have a potential therapeutic role in the treatment of chronic wounds [52]. Indeed, the treatment of HaCaT cells with AM reduced TGFß signalling in such a way that the phosphorylation of Smad2 or Smad3 seriously decreased. Reduction of TGFß signalling has been associated with the loss of the cell-cycle arrest that TGFß elicits in epithelial cells [38]. In pancreatic cancer cell lines in fact, attenuation of TGFß signalling has been pointed to as the cause for their failure to achieve proper cell cycle arrest in response to TGFß [38]. So, in our case the attenuation of TGFß-induced phosphorylation of Smad2 and Smad3 could explain the effect of AM on chronic wounds. The lack of TGFß signalling or Smad3 has been suggested as a way to uncouple TGFß signalling and wound healing [19]. Paradoxically, although a Smad2 KO is not obtainable, even heterozygosity for Smad2 does not affect the epithelialisation as heterozygosity for Smad3 does, suggesting that Smad2 is not involved in the same aspects of wound healing as Smad3 [27]. We tried to understand the possible cause of attenuation of Smad2 and Smad3 phosphorylation in response to TGFß. However, we have not seen any differences either in the concentration of TGFß receptors I/II or in the expression of Smad7 (data not shown) [53] upon long treatment with AM. Indeed, more research must be conducted to throw light on this issue. The Smad pathway stimulated by TGFß has also been involved in the production of fibrosis and inflammation in response to TGFß. Thus, interfering with TGFß signalling may be a good way of interfering with fibrosis and improving the evolution of wound healing [20]. Indeed, the application of AM is able to ameliorate fibrosis in different experimental models [54–58]. It will be very useful to see whether the application of AM is able to reduce fibrosis and inflammation in its application to chronic wounds.

Upregulating the expression of CDKN2B (p15) and CDKN1A (p21) proteins had been directly related to the TGFß-induced growth arrest of epithelial cells [26]. In our hands, the attenuation of TGFß dependent phosphorylation of both Smad2 and Smad3 by AM is linked to the expression attenuation of CDKN2B (p15) and CDKN1A (p21). However, the attenuating effect of AM on the phosphorylation of Smad2/3 did not have a general attenuation effect over all TGFß-regulated genes. In fact, the simultaneous treatment of keratinocytes with AM and TGFß only exhibit a clear attenuation of *CDKN2B (p15)* expression. Strikingly, when the cells were incubated for a longer time with AM (24 h), AM inhibited TGFß-induced up-regulation of both*CDKN2B (p15)* and *CDKN1A (p21)* genes, suggesting the need to condition the cells for a longer time with AM to prevent TGFß-induced *CDKN1A (p21)* expression. In addition, the data obtained for protein expression for these two genes was fully consistent with these data, suggesting that the effect of AM is related to gene regulation control but not to postranslational regulation or the stability of proteins. In the presence of AM, TGFß was unable to exert its cell cycle arrest. AM attenuates TGFß-induced Smad2/3 phosphorylation and *CDKN2B (p15)* and *CDKN1A (p21)* expression, and this is related to cell cycle regulation [38]. So the presence of AM counteracts the cell cycle arrest induced by TGFß on keratinocytes releasing them from the brake imposed by TGFß.

When looking at the expression data of other TGFß-regulated genes, we observed that not all were negatively affected by treatment with AM. We found that *ITGB6*, *TMEPAI*, *SNAI2* and *PAI1* were upregulated in primary keratinocytes stimulated for 24 h with AM, for all genes the additional treatment with TGFß increased the expression further. *ITGB6* and *TMEPAI* had been both related to enhanced cell migration [59–61]. PAI-1 is the major inhibitor of the serine proteases, the urokinase-type plasminogen activator (uPA) and the tissue type plasminogen activator (tPA). Elevated levels of PAI-1 protect extracellular matrix (ECM) proteins from proteolytic degradation, thus helping to accelerate wound healing [62, 63]; PAI-1 stimulates migration, facilitating the re-epithelialisation of the wound bed and promoting successful cellular attachment to the ECM [64, 65]. PAI-1 is a key molecule in the rapid attachment/detachment events required for cell migration [63, 66, 67]. Finally, keratinocytes from PAI-1 knockout mice had a significant wound-healing defect and the addition of active PAI-1 protein to *PAI-/-* keratinocytes rescued the migratory phenotype [66]. On the other hand, *SNAI2* belongs to the Snail family of zinc finger transcription factors. It plays an important role in Epithelial to Mesenchymal Transition (EMT) during wound re-epithelialisation. SNAI2 expression is elevated in keratinocytes at the margins of healing wounds in mice in vivo [68] and in keratinocytes migrating from mouse skin explants *ex vivo*, and in human keratinocytes at wound margins in vitro [69]. *SNAI2*-deficient mice have severely retarded motility of epidermal keratinocytes, suggesting that it is essential for wound re-epithelialisation [69]. We found that *SNAI2* and *PAI1* were upregulated in primary keratinocytes stimulated for 24 h with AM; for both genes the additional treatment with TGFß increased further the expression.

It is well known that mitogen-activated protein (MAP) kinase family members such as ERK1/2 and JNK are important for cell migration [70–74]. JNK signalling is critical in the movement of epithelial sheets, wound healing, apoptosis, cell survival and tumour development [75]. In vitro, mammalian JNKs efficiently phosphorylate c-Jun on two serine residues (Ser63 and Ser73) in the amino-terminal domain of the protein. This phosphorylation correlates well with c-Jun activation [76]. Our results showed that HaCaT cells stimulated with AM for 24 h exhibited an increased expression of *c-JUN*. Also, AM induced the phosphorylation of JNK kinase; moreover, c-Jun upregulation was enhanced when cells were co-stimulated with TGFß, either simultaneously or sequentially. We observed a similar phenomenon in primary keratinocytes: JNK phosphorylation was maximal when cells were treated simultaneously with AM and TGFß. Additionally, Mv1Lu cells upregulated and phosphorylated c-Jun in response to AM, and that correlates well with JNK1 activation by AM (data not shown). JNK1 is a positive regulator of c-Jun, it contributes to its phosphorylation, upregulation and stabilization [17, 18]. Furthermore, the use of SP600125 attenuated the expression and phosphorylation of c-Jun in response to AM. The phosphorylation of JNK1 by AM may be also responsible for the upregulation and activation of c-Jun protein in HaCaT cells and keratinocytes, that is increased further when TGFß is present. In fact, the expression of c-Jun protein was maximal when cells were stimulated with both AM and TGFß. Members of the AP1 family had been involved in keratinocyte migration. It is well known that AP1 transcription factors regulate the expression of various genes involved in the wound healing process [13, 14]. Upon the wounding of organotypic cultures of E17 rat skin, transient induction of *c-FOS* and *c-JUN* occurs [15]. Mice with keratinocyte-specific deletion of the *c-JUN* gene had delayed wound closure owing to impaired keratinocyte migration [16].

Re-epithelialisation involves migration and proliferation of keratinocytes to cover the wounded surface [77]. As the cells at the wound edge migrate into the injury site, keratinocytes are proliferating behind the actively migrating cells [78]. Importantly, the lack of Smad3 has a modification in the proliferation response of keratinocytes to TGFß that has consequences over wound healing increasing migration of keratinocytes. This effect can be seen in heterozygous mice for Smad3 [20]. Additionally, down regulation of Smad3 can accelerate wound healing in mouse palatal wound closure models [79]. TGFß signalling affects the migration of monocytes and neutrophils in Smad3 depleted mouse, however, no migration defects for keratinocytes were reported in such KO mice [20], implying that the impairment of TGFß signalling did not affect the cell migration of keratinocytes for wound healing. In the Mv1Lu wound healing scratch assay, AM induced the migration of cells. Moreover, the inhibition of Mv1Lu cell proliferation by MMC did not affect the wound healing properties suggesting that the neat contribution of AM to cell migration stimulation must be very important. However, when tested in HaCaT cells, AM promoted-migration was dependent on cell proliferation. Indeed the treatment of HaCaT cells with MMC prevented in vitro epithelialization. Additionally, the fact that AM membrane was able to antagonise TGFß effects on cell cycle control, suggest that AM, by antagonising the effects of TGFß on cell cycle regulation, may contribute to the epithelialisation observed in the chronic wounds upon AM treatment and suggest the importance of AM-induced cell proliferation for wound healing. Other inducers of cell migration also recruit the participation of c-Jun, for instance the Fibroin and Sericin activation of cell migration involves the local overexpression of c-Jun protein at the cells wound edge [42]. The usage of JNK1 inhibitors prevented AM-induced cell migration, and that correlated well with the lack of phosphorylation of JNK1/c-Jun or c-Jun upregulation in response to AM when SP600125 was present. A closer examination of the wound margins of the scratch wound healing assays showed a high expression of c-Jun of the cells engaged in the migratory front in both the AM stimulated cells and EGF stimulated cells. An AM-induced high expression of c-Jun at the wound border was prevented by inhibitors SP600125 and U0126, which is consistent with the fact that AM induced the activation of a signalling cascade that produced the phosphorylation of ERK1/2 and JNK1/2. It has been shown that TGFß is able to stimulate phosphorylation of p38 in epithelial cells [23, 80]. Although, phosphorylation of p38 kinase has also been associated with cell migration [41], it may not participate in the mechanism elicited by AM because SB203880 did not prevent either AM induced Mv1Lu or HaCaT cell migration nor c-Jun border expression in scratch wound healing experiments. We have done further research to expand the knowledge on the molecular mechanism underlying the AM-induction of migration to deeply understand AM healing properties [81]. Nevertheless, the fact that in mammalian cells, JNK is only phosphorylated in cells at the edge of the wound and inhibition of JNK pathway blocks migration and lamelipodia extension [82], suggests that AM induces a local increase in c-Jun in the patient wound border or both HaCaT and Mv1Lu scratch wound healing assay that is consistent with its effect on epithelialization. When wound borders were examined 5 and 15 days after AM application, a clear proliferation/migration was observed. Finally, our data clearly show that the consequence of the application of AM on the wound is a robust expression of c-Jun at the wound border, that is especially strong at the *stratum basale* of the epidermis coinciding with the keratinocyte tongue, the area where the migration of keratinocyte to close the wound is produced.

Briefly, in big massive chronic wounds, the high concentrations of TGFß induced by inflammation, may be the cause that prevents reepithelialisation, even when wound cavity has been filled by granulation tissue. Thus, the effect of AM on the modulation of TGFß responses in keratinocytes that favours proliferation together with AM-induced keratinocyte migration could allow chronic wounds to move on from their non-healing state and progress into epithelialization. It would be important if the factors responsible for the described effects of AM in this study were isolated and characterized in the future. Also, understanding the molecular mechanisms involved in regulating TGFß signalling during wound healing may provide important insights into how its deregulation may contribute to impaired wound healing [19]. Currently no therapies are available that target the TGFß signalling pathway to improve wound healing outcome [19]. We have shown that the application of Amniotic Membrane is able to promote healing in chronic wounds by modifying the genetic program induced by TGFß, stimulating keratinocyte proliferation and migration.

## Supporting Information

S1 Fig TGFß was able to elicit responses in human primary keratinocytes. Primary keratinocytes were grown and stimulated with TGFß for the indicated times. Proteins analysed are indicated. ß-actin was used as a loading control. This experiment was performed at least three times. A representative result is shown.
(TIF)

S2 Fig AM induced motility in Mv1Lu cells and the expression of c-Jun at the migratory front. **(A),** Wound healing scratch assay was performed in Mv1Lu cells in the presence of AM, EGF or combinations of AM with different inhibitors. Cells forming a confluent epithelium were wounded and immediately treated as indicated for 29 h. Representative pictures were taken at the beginning of the treatment and 29 h later. (**B**), Mv1Lu cells were treated with AM for the indicated times in the absence or presence of SP600125 and PD98059 inhibitors. Protein extracts were analysed by western blot for the indicated proteins. ß-actin was used as a loading control. (**C**), Treatment of Mv1Lu cells with AM cause the cells to express c-Jun at the migratory front. Wound healing scratch assay was treated with AM, EGF or combinations of AM with different inhibitors. Mv1Lu were wounded and treated for 25 h. Cells were fixed and immunostained for c-Jun. Images of c-Jun fluorescence were converted into pseudo-colour to show the intensity of c-Jun staining. Colour rainbow scale represents fluorescence intensity for c-Jun. Co-staining with phalloidin and Hoechst-33258 was used to show the cell structure and nuclei, respectively. Images were taken by confocal microscopy using a Zeiss 510 LSM confocal microscope. These experiments were repeated at least three times. A representative result is shown. Scale Bars 100 μm.
(TIF)

S3 Fig Treatment with MMC did not prevent either AM induced motility or c-Jun expression at the migratory front. **(A),** Wound healing scratch assay was performed in Mv1Lu in the presence of MMC cells in the presence of AM, EGF or combinations of AM with different inhibitors. Cells forming a confluent epithelium were treated with MMC, wounded and immediately treated for 26 h as indicated. Representative pictures were taken at the beginning of the treatment and 26 h later. (**B**), Stimulation with AM of MMC pretreated Mv1Lu cells cause the c-Jun expression at the migratory front. Wound healing scratch assay was treated with AM, EGF or combinations of AM with different inhibitors. Mv1Lu were wounded and treated for 25 h. Cells were fixed and immunostained for c-Jun. Images of c-Jun fluorescence were converted into pseudo-colour to show the intensity of c-Jun staining. Colour rainbow scale represents fluorescence intensity for c-Jun. Co-staining with phalloidin and Hoechst-33258 was used to show the cell structure and nuclei, respectively. Images were taken by confocal microscopy using a Zeiss 510 LSM confocal microscope. These experiments were done at least three times. A representative result is shown. Scale Bars 100 μm.
(TIF)
