# Correction: Amniotic Membrane Modifies the Genetic Program Induced by TGFß, Stimulating Keratinocyte Proliferation and Migration in Chronic Wounds

**DOI:** 10.1371/journal.pone.0141026

**Published:** 2015-10-15

**Authors:** Antonia Alcaraz, Anna Mrowiec, Carmen Luisa Insausti, Ángel Bernabé-García, Eva María García-Vizcaíno, María Concepción López-Martínez, Asunción Monfort, Ander Izeta, José María Moraleda, Gregorio Castellanos, Francisco José Nicolás

There are errors in the Funding section. The correct funding information is as follows: Plan Estatal I+D+i and Instituto de Salud Carlos III-Subdirección General de Evaluación y Fomento de la Investigación (Grant no.: PI13/00794) http://www.isciii.es/ Fondos FEDER (ERDF funds) http://ec.europa.eu/regional_policy/es/funding/erdf/.
