# Supplementary figures and images for: Amniotic Membrane Modifies the Genetic Program Induced by TGFß, Stimulating Keratinocyte Proliferation and Migration in Chronic Wounds
Source: PLoS One. 2015 Aug 18;10(8):e0135324. doi: 10.1371/journal.pone.0135324 (PMC4540284; doi:10.1371/journal.pone.0135324)

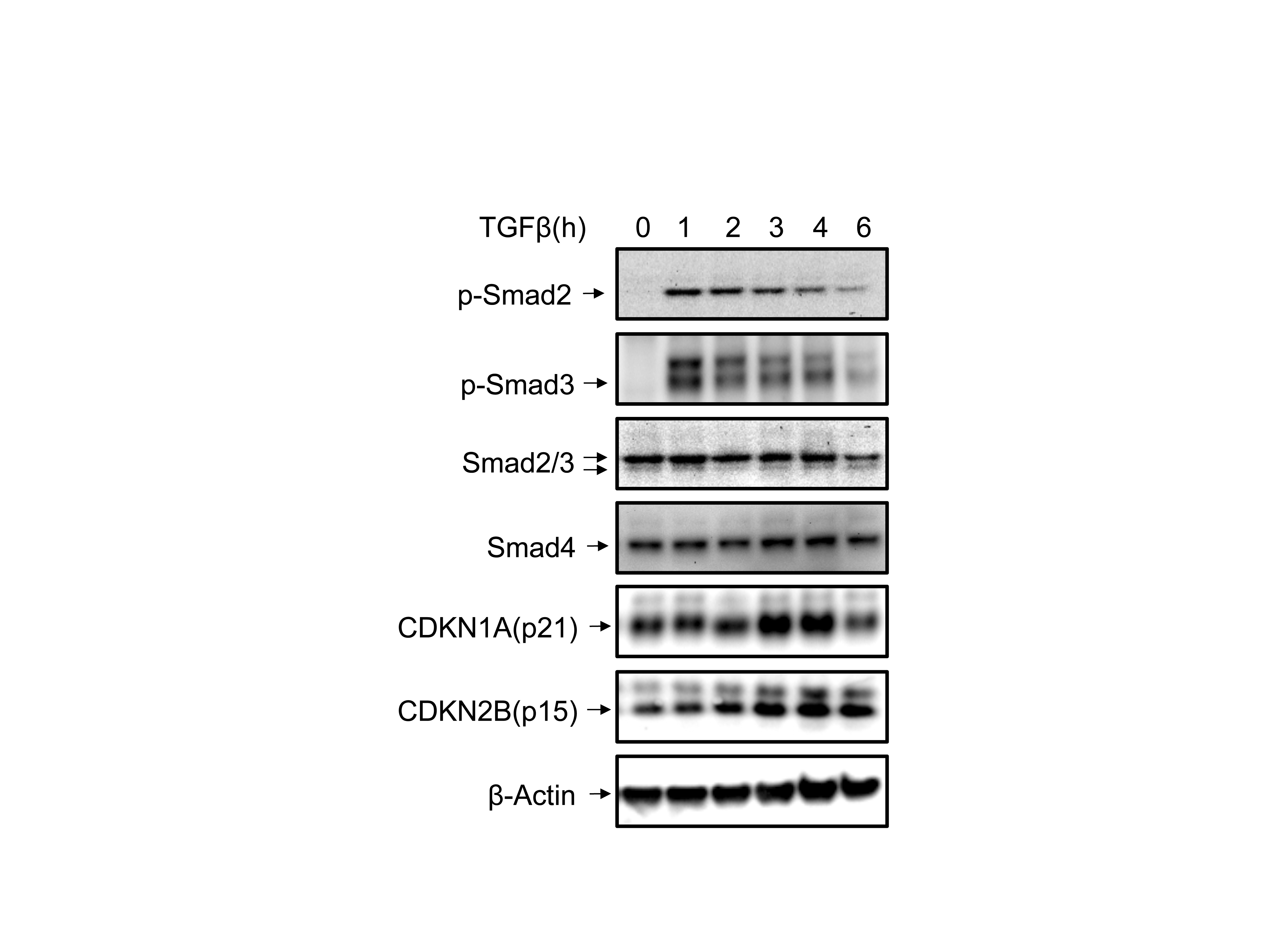

Supplement: S1 Fig — Primary keratinocytes were grown and stimulated with TGFß for the indicated times. Proteins analysed are indicated. ß-actin was used as a loading control. This experiment was performed at least three times. A representative result is shown. (TIF) [file pone.0135324.s001.tif]

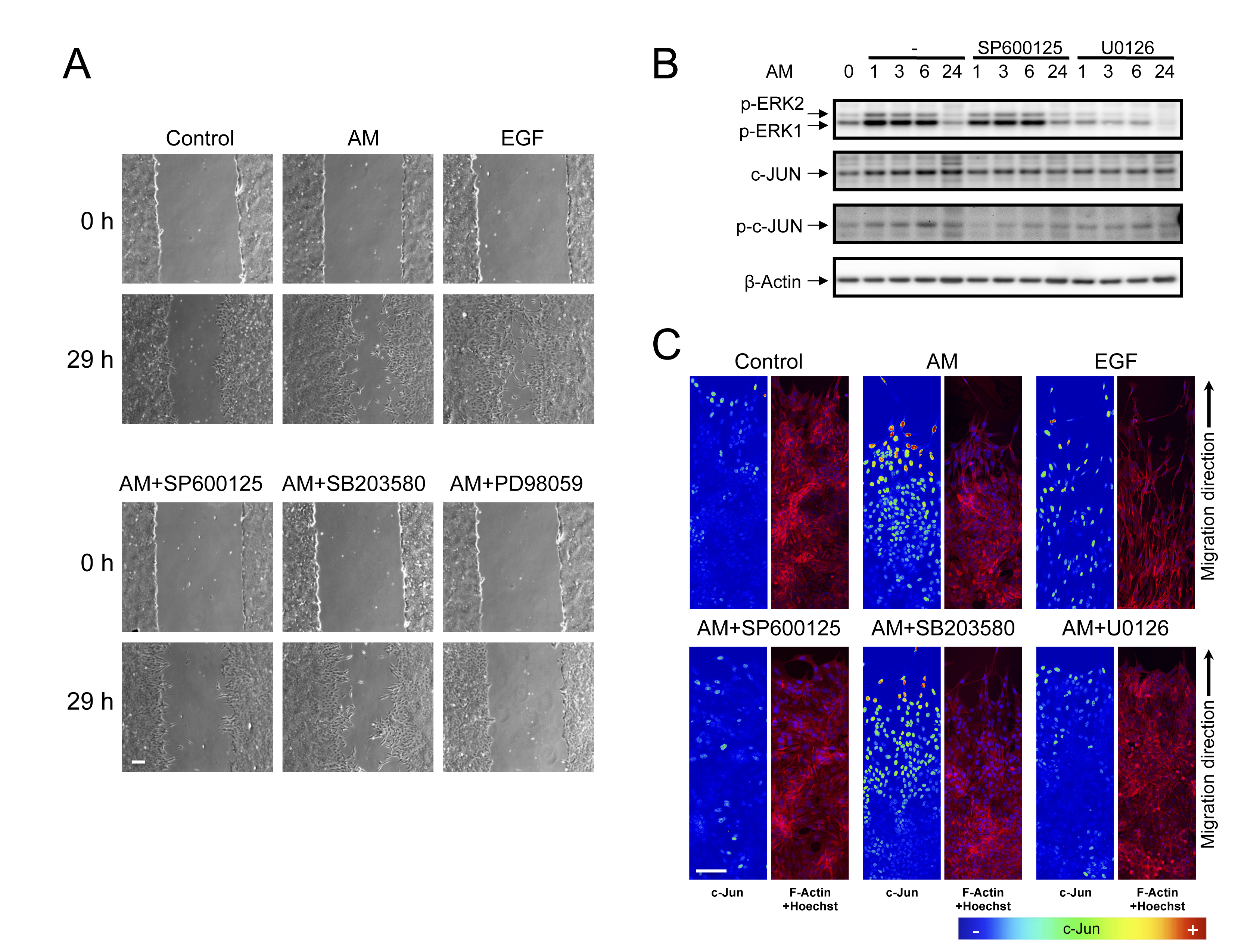

Supplement: S2 Fig — (A), Wound healing scratch assay was performed in Mv1Lu cells in the presence of AM, EGF or combinations of AM with different inhibitors. Cells forming a confluent epithelium were wounded and immediately treated as indicated for 29 h. Representative pictures were taken at the beginning of the treatment and 29 h later. (B), Mv1Lu cells were treated with AM for the indicated times in the absence or presence of SP600125 and PD98059 inhibitors. Protein extracts were analysed by western blot for the indicated proteins. ß-actin was used as a loading control. (C), Treatment of Mv1Lu cells with AM cause the cells to express c-Jun at the migratory front. Wound healing scratch assay was treated with AM, EGF or combinations of AM with different inhibitors. Mv1Lu were wounded and treated for 25 h. Cells were fixed and immunostained for c-Jun. Images of c-Jun fluorescence were converted into pseudo-colour to show the intensity of c-Jun staining. Colour rainbow scale represents fluorescence intensity for c-Jun. Co-staining with phalloidin and Hoechst-33258 was used to show the cell structure and nuclei, respectively. Images were taken by confocal microscopy using a Zeiss 510 LSM confocal microscope. These experiments were repeated at least three times. A representative result is shown. Scale Bars 100 μm. (TIF) [file pone.0135324.s002.tif]

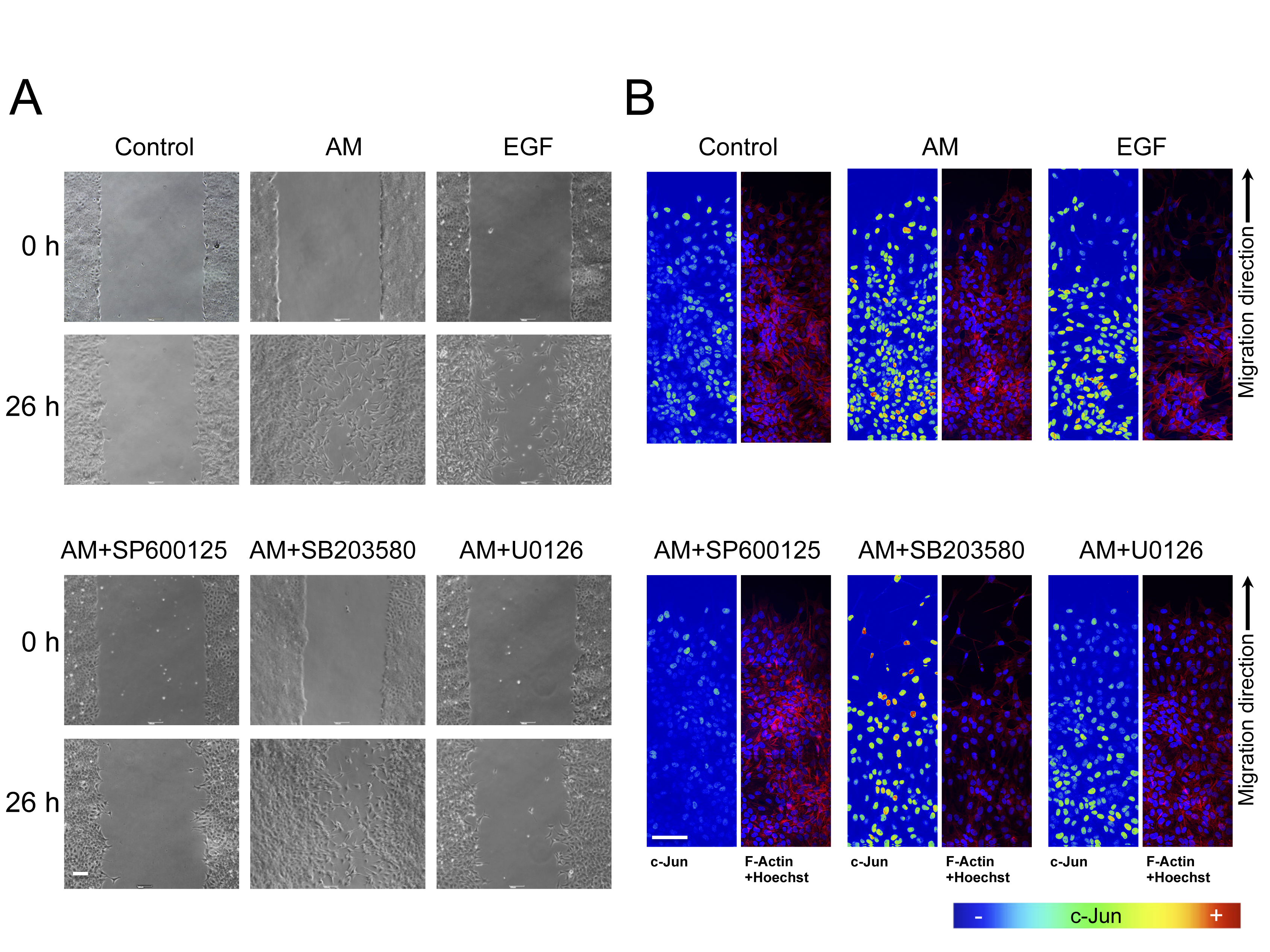

Supplement: S3 Fig — (A), Wound healing scratch assay was performed in Mv1Lu in the presence of MMC cells in the presence of AM, EGF or combinations of AM with different inhibitors. Cells forming a confluent epithelium were treated with MMC, wounded and immediately treated for 26 h as indicated. Representative pictures were taken at the beginning of the treatment and 26 h later. (B), Stimulation with AM of MMC pretreated Mv1Lu cells cause the c-Jun expression at the migratory front. Wound healing scratch assay was treated with AM, EGF or combinations of AM with different inhibitors. Mv1Lu were wounded and treated for 25 h. Cells were fixed and immunostained for c-Jun. Images of c-Jun fluorescence were converted into pseudo-colour to show the intensity of c-Jun staining. Colour rainbow scale represents fluorescence intensity for c-Jun. Co-staining with phalloidin and Hoechst-33258 was used to show the cell structure and nuclei, respectively. Images were taken by confocal microscopy using a Zeiss 510 LSM confocal microscope. These experiments were done at least three times. A representative result is shown. Scale Bars 100 μm. (TIF) [file pone.0135324.s003.tif]
